# A comparative study of circulating tumor cell isolation and enumeration technologies in lung cancer

**DOI:** 10.1002/1878-0261.13705

**Published:** 2024-08-06

**Authors:** Volga M Saini, Ezgi Oner, Mark P. Ward, Sinead Hurley, Brian David Henderson, Faye Lewis, Stephen P. Finn, Gerard J. Fitzmaurice, John J. O'Leary, Sharon O'Toole, Lorraine O'Driscoll, Kathy Gately

**Affiliations:** ^1^ Thoracic Oncology Research Group, Trinity Translational Medicine Institute St James's Hospital Dublin Ireland; ^2^ Department of Clinical Medicine, School of Medicine Trinity College Dublin Ireland; ^3^ Trinity St. James's Cancer Institute Trinity College Dublin Ireland; ^4^ Department of Obstetrics and Gynaecology, School of Medicine Trinity College Dublin Ireland; ^5^ Department of Histopathology and Morbid Anatomy, School of Medicine Trinity College Dublin Ireland; ^6^ Department of Cardiothoracic Surgery St James's Hospital Dublin Ireland; ^7^ School of Pharmacy and Pharmaceutical Sciences Trinity College Dublin Ireland; ^8^ Trinity Biomedical Sciences Institute Trinity College Dublin Ireland

**Keywords:** CellMag™, circulating tumor cells, EasySep™, non–small cell lung cancer, Parsortix®, RosetteSep™

## Abstract

Circulating tumor cells (CTCs) have potential as diagnostic, prognostic, and predictive biomarkers in solid tumors. Despite Food and Drug Administration (FDA) approval of CTC devices in various cancers, the rarity and heterogeneity of CTCs in lung cancer make them technically challenging to isolate and analyze, hindering their clinical integration. Establishing a consensus through comparative analysis of different CTC systems is warranted. This study aimed to evaluate seven different CTC enrichment methods across five technologies using a standardized spike‐in protocol: the CellMag™ (EpCAM‐dependent enrichment), EasySep™ and RosetteSep™ (blood cell depletion), and the Parsortix® PR1 and the new design Parsortix® Prototype (PP) (size‐ and deformability‐based enrichment). The Parsortix® systems were also evaluated for any differences in recovery rates between cell harvest versus in‐cassette staining. Healthy donor blood (5 mL) was spiked with 100 fluorescently labeled EpCAM^high^ H1975 cells, processed through each system, and the isolation efficiency was calculated. The CellMag™ had the highest recovery rate (70 ± 14%), followed by Parsortix® PR1 in‐cassette staining, while the EasySep™ had the lowest recovery (18 ± 8%). Additional spike‐in experiments were performed with EpCAM^moderate^ A549 and EpCAM^low^ H1299 cells using the CellMag™ and Parsortix® PR1 in‐cassette staining. The recovery rate of CellMag™ significantly reduced to 35 ± 14% with A549 cells and 1 ± 1% with H1299 cells. However, the Parsortix® PR1 in‐cassette staining showed cell phenotype‐independent and consistent recovery rates among all lung cancer cell lines: H1975 (49 ± 2%), A549 (47 ± 10%), and H1299 (52 ± 10%). Furthermore, we demonstrated that the Parsortix® PR1 in‐cassette staining method is capable of isolating heterogeneous single CTCs and cell clusters from patient samples. The Parsortix® PR1 in‐cassette staining, capable of isolating different phenotypes of CTCs as either single cells or cell clusters with consistent recovery rates, is considered optimal for CTC enrichment for lung cancer, albeit needing further optimization and validation.

## Graphical abstract

Lung cancer cells were spiked into donor blood to evaluate the recovery rates of the following circulating tumor cell (CTC) enrichment technologies: CellMag™, EasySep™, RosetteSep™, Parsortix® PR1, and Parsortix® Prototype systems. Each method's advantages and disadvantages are described. The Parsortix® PR1 in‐cassette staining method showed cell phenotype‐independent and consistent recovery rates across cell lines and isolated single CTCs and clusters from patient blood.

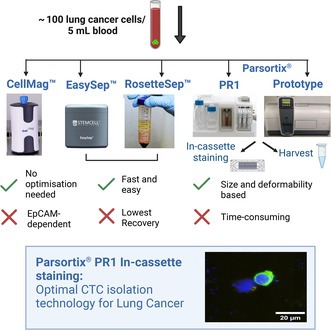

**Abbreviations**
CCRT: concurrent chemoradiotherapy
CK: cytokeratin
CK19: cytokeratin 19
CTC: circulating tumor cell
DAPI: 4′,6‐diamidino‐2‐phenylindole
EDTA: ethylenediaminetetraacetic acid
EMT: epithelial–mesenchymal transition
EpCAM: epithelial cellular adhesion molecule
EV: extracellular vesicle
FDA: Food and Drug Administration
NSCLC: non–small cell lung cancer
PanCK: pan cytokeratin
PP: Parsortix® Prototype
RR: recovery rate

## 1 Introduction

Circulating tumor cells (CTCs) are shed into the blood from either primary or metastatic tumors and can provide an important snapshot of tumor heterogeneity and metastatic potential. Recent advances in the sensitivity of cellular and molecular detection technologies have increased our understanding of the molecular characteristics and timing of dissemination of CTCs during cancer progression. Single‐cell, multi‐omics technologies have shown that CTCs are heterogeneous, disseminate early, and represent a subpopulation of the primary tumor responsible for disease relapse [1]. CTC enumeration has been widely used as a prognostic biomarker to monitor treatment response and predict disease relapse. In non–small cell lung cancer (NSCLC), CTC counts are low but have been shown to be prognostic in early‐stage disease [2, 3, 4]. Interestingly, several studies have shown that blood collected from the pulmonary vein during surgery contains higher numbers of CTCs than blood from the peripheral vein [5, 6, 7, 8, 9]. Detection rates in metastatic NSCLC can be as low as 1–10 CTC·mL^−1^ blood with slightly higher rates in small cell lung cancer (SCLC) [10]. In advanced disease, changes in CTC counts in response to chemotherapy [11] or EGFR tyrosine kinase inhibitors [12] have highlighted their potential as predictive biomarkers. The prognostic value of CTC counts in patients with stage IV NSCLC has been confirmed by a comprehensive meta‐analysis using data from seven different CTC centers [13]. Their predictive and prognostic impact is further increased when combined with other blood constituent characteristics, such as extracellular vesicle (EV) miRNA cargo [14] or EV protein expression [15], for concurrent chemoradiotherapy (CCRT) treatment monitoring and overall survival, respectively.

Despite the presence of CTCs indicating a poor prognosis in patients with NSCLC, their scarcity in blood compared to other cell types has hindered the translation of CTC molecular profiling to the clinic. This is a significant challenge particularly in early‐stage disease where their identification and characterization could offer the most benefit for patients, helping to guide treatment and disease management. However, the field of CTC isolation is a rapidly evolving one and over the past decade there has been a surge in new CTC isolation devices and downstream workflows for molecular profiling. Current technologies are primarily based on immunoaffinity (positive or negative enrichment strategies) or biophysical properties. Although two devices are Food and Drug Administration (FDA) approved for CTC enrichment, neither are approved in NSCLC. The CellSearch® CTC Test (Menarini‐Silicon Biosystems, Bologna, Italy) is approved in breast, colorectal, and prostate cancer [16, 17, 18]. In 2022, the Parsortix® PC1 System (ANGLE plc, Guildford, UK) received FDA clearance for capturing and harvesting of CTCs from the blood of metastatic breast cancer patients enabling subsequent user‐validated analysis [19].

Currently the assessment of CTCs has not translated into the routine clinical management of patients with NSCLC. Reproducibility and sensitivity remain a challenge as do issues with manual processes and low recovery rates [20, 21, 22, 23]. Here we evaluate the recovery rates of five different CTC enrichment technologies including: the CellMag™ system which uses a manual immunomagnetic (ferrofluid technology) enrichment of epithelial cells using the epithelial cellular adhesion molecule (EpCAM) and is based on the same principle as the CellSearch®; EasySep™ targets cells for either removal (negative selection via CD45 depletion) or positive selection using antibody complexes directed to specific cell surface antigens; RosetteSep™ is an immunodensity procedure that isolates unlabeled CTCs using an antibody cocktail to deplete monocytes along with erythrocytes from whole blood; Parsortix® PR1 enables the separation and capture of cells present in blood based on their size and deformability [20]; and the Parsortix® Prototype (PP) which is ANGLE plc's new design currently undergoing beta testing and utilizes the same isolation method as the PR1.

## 2 Materials and methods

### 2.1 Preparation of cells prior to spiking

#### 2.1.1 Cell culture

The NSCLC cell line NCI‐H1975 (RRID: CVCL_1511) was used for initial spike‐in experiments to compare the seven different CTC enrichment methods across five technologies. A variant of this cell line expressing green fluorescent protein (H1975‐GFP) and stained with the CellTracker™ Green CMFDA (5‐chloromethylfluorescein diacetate) dye was also utilized to ensure the detectability of all cells. A549 (RRID: CVCL_0023) and NCI‐H1299 (RRID: CVCL_0060) cell lines were used for additional spike‐in experiments to compare the recovery rates between the CellMag™ and Parsortix® PR1 in‐cassette staining methods. H1975 and A549 are lung adenocarcinoma cell lines, while H1299 is a large cell carcinoma cell line. These three cell lines were chosen as they have differential EpCAM expression. The GFP transfected variant of the H1975 cell line and the A549 cell line were kind gifts from S. Gray and M. Barr from the Trinity Translational Medicine Institute, St. James's Hospital, Ireland, respectively. All cell lines were originally purchased from the American Type Culture Collection (ATCC, Manassas, VA, USA) and authenticated using short tandem repeat (STR) analysis within the past 3 years and regularly checked for the absence of mycoplasma. H1975, H1975‐GFP, and H1299 cell lines were cultured in RPMI 1640 GlutaMAX™ (#618700510; Gibco, Paisley, UK) with 10% fetal bovine serum (FBS) (#F7524; Sigma‐Aldrich, St. Louis, MO, USA) and 1% penicillin/streptomycin (P/S; #15140‐122; Gibco). A549 cells were cultured in Nutrient Mixture F‐12 Ham (#N4888; Sigma‐Aldrich) with 10% FBS, 1% P/S, and 1% l‐glutamine (#25030081; Gibco). All cell lines were cultured in a humidified atmosphere at 37 °C and 5% CO_2_.

#### 2.1.2 Reagents and solutions

- 0.5 m Ethylenediaminetetraacetic acid (EDTA) (50 mL): 9.305 g EDTA (disodium salt, molecular weight = 372.4 g·mol^−1^) and 40 mL deionized water. Adjust pH to 8 by adding sodium hydroxide. Make up to 50 mL with deionized water.
- Spike in buffer (10 mL): 40 μL 0.5 m EDTA, 2 mL 5% bovine serum albumin (BSA), and 8 mL Dulbecco's phosphate‐buffered saline (DPBS) without Ca^2+^ and Mg^2+^ (SH30028.02; Cytiva, South Logan, UT, USA).
- EasySep™ buffer (50 mL): 49 mL DPBS without Ca^2+^ and Mg^2+^, 1 mL FBS, and 100 μL 0.5 m (500 mm) EDTA.
- Recommended medium for RosetteSep™ (DPBS with 2% FBS, 500 mL): Add 10 mL FBS to 490 mL DPBS.

#### 2.1.3 Cell labelling using CellTracker™ dye

Cells were prelabeled with the CellTracker™ Green CMFDA dye (Catalog #C7025; Thermo Fisher Scientific, Eugene, OR, USA) prior to spike‐in experiments to facilitate detection and enumeration. Cells were at approximately 80–90% confluence on the day of staining. After trypsinization and centrifugation, the cell pellet was resuspended in 4 mL of growth media. Of this cell suspension, 2 mL was transferred to another 15 mL tube and centrifuged at 400 ***g*** for 3 min. The below steps were then performed with the light off in a sterile biosafety cabinet. The cell pellet was then resuspended in 2 mL of the respective cell media containing 2 μL of 5 mm CellTracker™ Green CMFDA dye stock solution (1 : 1000 dilution, final concentration: 5 μm) and 4 μL of 1 mg·mL^−1^ Hoechst 33342 dye (1 : 500 dilution) (#H3570; Life Technologies, Eugene, OR, USA) and resuspended thoroughly using a low binding pipette tip. Precautions were taken to protect the now‐fluorescently labeled cells from the light. The stained cells (in 15 mL tube) were covered with tinfoil and placed in a humidified incubator at 37 °C with 5% CO_2_ for 30 min. After incubation, the stained cell suspension was centrifuged at 400 ***g*** for 3 min and supernatant removed. The labeled cells were washed by resuspending the cell pellet in 2 mL DPBS and then centrifuged at 400 ***g*** for 3 min. The supernatant was removed, and the cell pellet was resuspended in 1 mL of spike‐in buffer before counting. Spike‐in buffer was made fresh on the day of every experiment.

#### 2.1.4 Cell counting and serial dilution

Prior to serial dilution, cell counting was performed manually with an inverted microscope using a hemocytometer and trypan blue stain. The cell number was calculated by counting a total of eight squares (four outer corner squares on both top and bottom grids) of the hemocytometer each time. After resuspending the cell pellet in 1 mL of spike‐in buffer, cell counting was performed once if the cells in the hemocytometer were evenly distributed and each corner square contained 10–40 cells. Otherwise, cell counting was repeated by readjusting the spike‐in buffer volume accordingly and mixing thoroughly with trypan blue.

**Fig. 1:**
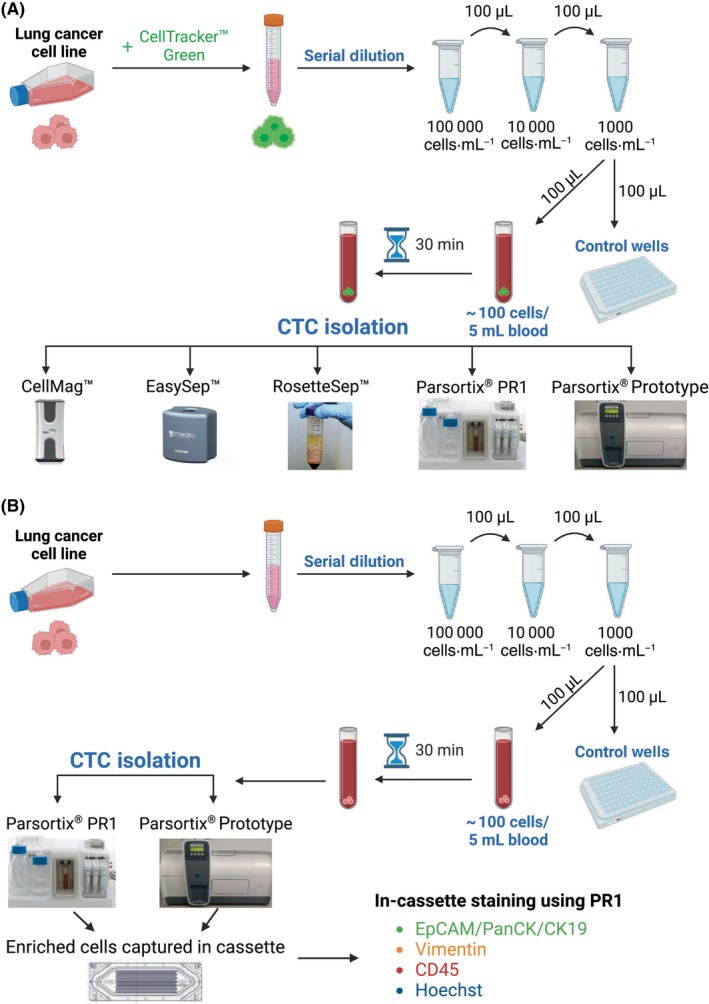
Overview of spike‐in experiment and comparison of circulating tumor cell (CTC) isolation systems. (A) Lung cancer cells were labeled with CellTracker™ Green CMFDA dye, and a cell suspension of 1000 cells·mL^−1^ was made. Of this fluorescently labeled cell suspension (containing approximately 100 cells), 100 μL were added to both 5 mL of healthy donor blood and each control well (at least two control wells). The spiked blood sample was incubated for 30 min before processing through the different CTC isolation systems: CellMag™, EasySep™, RosetteSep™, Parsortix® PR1, and Parsortix® Prototype (PP). (B) In order to compare the recovery rate of in‐cassette staining between the PR1 and PP systems, a similar protocol was followed using unlabeled cells. The enriched cells captured in the cassette were fixed and stained using the Parsortix® PR1 with Alexa Fluor (AF)‐conjugated antibodies against Cytokeratins (CK 4, 5, 6, 8, 10, 13, 18, and 19) and EpCAM (AF488, green), Vimentin (AF546, yellow), CD45 (AF647, red), and Hoechst nuclear stain (blue). This figure was created with BioRender.com.

Serial dilution was then performed using spike‐in buffer to create a cell suspension with a final concentration of approximately 1000 cells·mL^−1^ (Fig. 1). This suspension was stored in a humidified incubator at 37 °C with 5% CO_2_ until further use (maximum 15–20 min). Only low binding pipette tips and Eppendorf tubes were used in this process to minimize cell loss.

#### 2.1.5 Control wells

The serial dilution process described before is prone to error and it is difficult to determine exactly how many cells are being spiked into the blood. To overcome this issue, we included “control wells” to estimate how many cells were spiked (on average) into blood before processing through each CTC enrichment technology. The experimental replicates of each CTC enrichment technology were conducted on different days. In each spike‐in experiment (Fig. 1), 100 μL of the cell suspension (approximately containing 100 cells) was first resuspended and then pipetted into the 5 mL healthy donor blood sample for CTC isolation as described next. Second, 100 μL of the same cell suspension was resuspended and then pipetted into 2–4 control wells. The average number of cells counted from these control wells was estimated to be the approximate number of cells spiked into the blood before CTC isolation and was used to calculate the recovery rate for each system (Section 2.3).

For the control wells and other steps which included cell harvest, the well surface was coated with poly‐l‐lysine (#P4707; Sigma‐Aldrich) to improve cell adhesion. Each well of a 96‐well plate was coated with 50 μL of the poly‐l‐lysine and left to incubate for 5 min with the lid on at room temperature. After removing the poly‐l‐lysine, the wells were allowed to dry without the lid for 30 min. The 100 μL of the cell suspension at approximately 1000 cells·mL^−1^ concentration was mixed thoroughly and then transferred into separate poly‐l‐lysine‐coated control wells using a low binding pipette tip. The 96‐well plate was placed in the incubator (37 °C, 5% CO_2_) for 1 h to allow the cells to settle before visualization.

### 2.2 Enrichment of spiked cells using different CTC isolation systems

Between January 2023 and May 2024, healthy donors were prospectively recruited to the CLuB liquid biopsy study at Trinity College Dublin. Ethical approval was obtained from the Trinity College Dublin, School of Medicine Research Ethics Committee (Ref. No. 230308) in compliance with GDPR and the Health Research Regulations 2018. The study was conducted in accordance with the precepts of the Code of Ethics of The World Medical Association (Declaration of Helsinki). All healthy donors provided their appropriate written informed consent to participate in the study prior to enrollment.

#### 2.2.1 CellMag™ Epithelial CTC Kit

The manual CellMag™ epithelial CTC kit (#9603), developed by Menarini Silicon Biosystems, operates on the same principle as the FDA‐approved CellSearch® system. However, the CellMag™ system is intended for research use only. This technology isolates circulating epithelial cells from whole blood by immunomagnetic positive enrichment. The CellMag™ kit consists of a ferrofluid‐based capture reagent that contains magnetic particles which are coated with anti‐EpCAM antibodies to capture epithelial cells in circulation. After enrichment, the captured cells are labeled with fluorescent reagents for identification. These fluorescent staining reagents include 4′,6‐diamidino‐2‐phenylindole (DAPI) (blue fluorescent DNA stain), anti‐CK‐PE (mouse monoclonal antibodies specific to cytokeratins conjugated to phycoerythrin) which stains epithelial cells and anti‐CD45‐APC (anti‐mouse CD45 monoclonal antibody conjugated to allophycocyanin) which stains leukocytes. In summary, the CellMag™ assay detects CTCs that are EpCAM+, CK+, and CD45−.

The CellMag™ Epithelial CTC Kit and the CellMag™ magnet were used to isolate spiked cells according to the manufacturers protocol (Fig. 1A). The only adjustment to the kit's protocol was that we included an additional centrifugation step before vortexing to obtain a more enriched population. The detailed protocol is listed in Appendix S1. Healthy donor blood was collected in a CellSave Preservative tube. After precoating a serological pipette with 1% BSA, 5 mL of this blood was transferred to a conical tube (containing spiked‐in cells stained with CellTracker™) and left to incubate at room temperature for 30 min before being processed through the CellMag™ system. Briefly, the sample was centrifuged at 800 ***g*** for 10 min for plasma separation. After plasma removal, 150 μL of capture enhancement reagent and anti‐EpCAM ferrofluid were added. Magnetic separation was then performed (placing the sample in CellMag™ magnet) in order to isolate the target cells. The negative fraction of the sample was aspirated using the kit's standardized aspiration tool. The target cells were washed again, and the supernatant was aspirated using the syringe as described earlier. The enriched target cells were then permeabilized and stained using the reagents included in the kit. The target cells were washed again, and the fluid and unlabeled cells were removed by aspiration. Finally, the enriched target cells were fixed, resuspended in CellMag™ buffer, and transferred to one well of the 96‐well plate. The 96‐well plate was placed in the incubator (37 °C, 5% CO_2_) for 1 h to allow the cells to settle before visualization.

#### 2.2.2 EasySep™ Direct Human CTC Enrichment Kit

The EasySep™ Direct Human CTC Enrichment Kit (#19657; StemCell Technologies) along with the “Big Easy” Magnet (#18001; StemCell Technologies, Vancouver, Canada) was used in this study to enrich cancer cells spiked into whole blood. This system enriches CTCs by immunomagnetic negative selection. This kit contains an antibody cocktail that targets surface markers (CD2, CD14, CD16, CD19, CD45, CD61, CD66b, and Glycophorin A) on hematopoietic cells and platelets for depletion. These unwanted cells, which are labeled with antibodies and Direct RapidSpheres™, can then be separated using an EasySep™ magnet.

The spiking of cultured cells into blood was performed as before and the isolation was performed as per the kits' instructions. The detailed protocol is listed in Appendix S2. We made various adjustments to the manufacturer's protocol based on the protocol used by Fankhauser et al. [24] to attain a highly pure, enriched population. Briefly, 5 mL of healthy donor blood was added to a 14 mL round bottomed tube containing the fluorescently labeled cells. Then, 250 μL of enrichment cocktail (provided in kit) was added and mixed slowly by inverting 10 times, followed by a 5‐min incubation at room temperature. RapidSpheres™ were vortexed for 30 s before 250 μL was added to the blood and mixed by inverting 10 times. EasySep™ buffer (Section 2.1.2) was then added to double the volume and mixed by inverting 10 times. The sample was placed in the “Big Easy” magnet and incubated at room temperature for 10 min. After the enriched cell suspension was transferred to a new 14 mL round bottomed tube, 250 μL of RapidSpheres™ was added to the sample and mixed by inverting 10 times. The tube was then placed in the Big Easy magnet and incubated at room temperature for 10 min. The entire enriched cell suspension was pipetted into a new 15 mL conical tube. The sample was centrifuged at 800 ***g*** for 5 min to pellet the cells. The supernatant was aspirated, and the cell pellet was resuspended in 200 μL of DPBS containing 0.2 μL of 1 mg·mL^−1^ Hoechst 33342 dye. The cell suspension was then transferred to a poly‐l‐lysine‐coated well of a 96‐well plate for the 1‐h incubation (37 °C, 5% CO_2_) followed by visualization. Hoechst dye was added to the enriched cell suspension to observe the level of leukocyte contamination.

#### 2.2.3 RosetteSep™ Human CD45 Depletion Cocktail

The RosetteSep™ Human CD45 Depletion Cocktail (#15112; StemCell Technologies) was used in this study to enrich cancer cells spiked into whole blood. This kit contains an antibody cocktail consisting of tetrameric antibody complexes that recognize CD45, CD66b, and glycophorin A, which crosslink unwanted hematopoietic cells to red blood cells, forming “immunorosettes”. These immunorosettes can be pelleted by centrifugation over a density gradient medium with a density of 1.077 g·mL^−1^, leaving enriched cells at the interface.

The spiked cells stained with CellTracker™ were isolated from the blood by following the kit's instructions (Appendix S3). Initially, 250 μL of RosetteSep™ Cocktail was added to the blood sample containing spiked cells and thoroughly mixed by pipetting and inverting the tube. The tube was then incubated at room temperature for 10 min. Subsequently, the blood sample was diluted with the recommended medium (Section 2.1.2) and mixed gently. Next, 15 mL of Ficoll‐Paque™ Plus (#17‐1440‐03; GE Healthcare, Uppsala, Sweden) density gradient medium was dispensed into a 50 mL SepMate™ tube (#85450; StemCell Technologies). The diluted blood sample was slowly pipetted on top of the Ficoll‐Paque™ Plus in the 50 mL tube and centrifuged at 1200 ***g*** for 10 min with the brake on. The supernatant was then transferred to a new standard tube and topped up with recommended medium to wash the enriched cells. The suspension was centrifuged at 300 ***g*** for 10 min with the brake off. The supernatant was discarded by aspiration, and the cell pellet was resuspended in 200 μL of DPBS and 0.2 μL of 1 mg·mL^−1^ Hoechst dye (diluted 1 : 1000). Finally, the cell suspension was transferred to a poly‐l‐lysine‐coated well in a 96‐well plate, which was placed in an incubator (37 °C, 5% CO_2_) for 1 h to allow the cells to settle before visualization.

#### 2.2.4 Parsortix® PR1

It is well established that cancer cells are more deformable and elastic than normal blood cells [25, 26]. The Parsortix® PR1 Cell Separation system utilizes these properties to preferentially isolate CTCs from blood. The system consists of the instrument and a disposable separation cassette. This microfluidic cassette contains steps that captures cells > 6.5 μm, well below the diameter of lung cancer cells [27]. Captured cells can be fixed, permeabilized, and stained with immunofluorescence markers (in‐cassette staining) and viewed under a fluorescent microscope. Alternatively, the captured cells can be flushed out and collected (cell harvest) for downstream analysis applications.

The spiked cells were recovered from the blood sample as per the manufacture's protocol (Appendix S4). Briefly, the “PX2_PF” protocol was run to “prime” the cassette. This step involves filling the cassette with a buffer solution, preparing the instrument to process a blood sample. Subsequently, the “PX2_S99F” protocol was run for blood processing and to capture the spiked cells in the cassette.

##### PR1 in‐cassette staining

In‐cassette staining was performed to fix and stain the captured cells in the cassette using the “PX2_Stain 3” protocol (Fig. 1B, Appendix S4.1). An optimized antibody recipe was developed for this staining protocol:

- Fixative: 360 μL DPBS and 40 μL formaldehyde solution (F8775; Sigma‐Aldrich).
- Antibody cocktail: 3.5 μL of each fluorescent‐conjugated primary antibody purchased from Santa Cruz Biotechnology (Dallas, TX, USA) (CD45 antibody [35‐Z6, Alexa Fluor 647, sc‐1178]; pan‐Cytokeratin antibody detecting cytokeratins 4, 5, 6, 8, 10, 13, and 18 [C11, Alexa Fluor 488, sc‐8018]; Cytokeratin 19 antibody [A53‐B/A2, Alexa Fluor 488, sc‐6278]; EpCAM antibody [HEA125, Alexa Fluor 488, sc‐59906], and Vimentin antibody [V9, Alexa Fluor 546, sc‐6260]) were added to 399 μL of permeabilization buffer Inside Perm (#130‐090‐477, Inside stain Kit; Miltenyi Biotec, Bergisch Gladbach, Germany), along with 3.5 μL of 1 mg·mL^−1^ Hoechst 33342 dye. The final dilution ratio of reagents in the buffer was 1 : 120 (v/v).

##### PR1 cell harvest

The cell harvesting procedure allows pulses of liquid to pass through the cassette in a reverse direction. This liquid detaches the captured cells and pushes them through the harvest line for collection in a collection tube. Before cell harvest, a “preharvest flush” was completed by running the “PX2_CT2” protocol to minimize the presence of residual blood cells (Appendix S4.2). After this process, the “PX2_H” protocol was run to harvest the captured cells in a volume of approximately 200 μL, in a low binding Eppendorf tube. Then, 0.2 μL of 1 mg·mL^−1^ Hoechst dye was added to this tube, and its contents were transferred to a poly‐l‐lysine‐coated well in a 96‐well plate. The plate was placed in an incubator (37 °C, 5% CO_2_) for 1 h to allow the cells to settle before visualization.

#### 2.2.5 Parsortix® Prototype

The Parsortix® Prototype (PP) is a new design of the Parsortix® PR1 and was beta tested in our laboratory. The main differences between both systems are that PP is a closed system (Fig. 1) and has a faster blood processing time than the PR1 (Table 1). Currently, the PP is only capable of performing cell harvest and cannot conduct in‐cassette staining.

**Table 1:** Summary of different CTC enrichment technologies tested.

	CellMag™	EasySep™	RosetteSep™	Parsortix® PR1	Parsortix® Prototype (PP)
Manufacturer	Menarini Silicon Biosystems	StemCell Technologies	StemCell Technologies	ANGLE plc	ANGLE plc
CTC isolation/detection method	Immunomagnetic, EpCAM dependent	Immunomagnetic, negative selection via CD45 depletion	Immunodensity, negative selection via CD45 depletion	Size‐ and deformability‐based microfluidic system	Size‐ and deformability‐based microfluidic system
CTC cluster‐capturing capability	N/A *CellSearch® system: Yes [64]	N/A	Yes [28, 63]	Yes [37, 65, 66]	N/A
Skill/complexity	Moderate	Low	Moderate	Low	Low
Advantages	Cost‐effective and straightforward protocol Standardized aspiration tool No need for antibody optimization	Fast and easy to use CTC isolation process	Results in unlabeled viable cells suitable for CTC culture Ability to capture CTC clusters	Recovery of heterogeneous population of CTCs Ability to capture CTC clusters	Recovery of heterogeneous population of CTCs Ability to capture CTC clusters Quicker separation process compared to PR1
Disadvantages	Only captures CTCs that express EpCAM Time‐consuming Not suitable for culture since cells are fixed	May inadvertently remove CTCs in clusters with blood cells	May inadvertently remove CTCs in clusters with blood cells	Only capturing CTCs above a certain size It is time‐consuming to analyze stained slides	Only capturing CTCs above a certain size It is time‐consuming to analyze stained slides No option for in‐cassette staining
“Hands‐on‐time”	5 h	2 h	2.5 h	45 min	35 min
Total time required (CTC isolation to enumeration)	6.5 h	3 h	3.5 h	Harvest: 2 h	Harvest: 1.25 h
				In‐cassette stain: 5 h	In‐cassette stain: 3.45 h

##### PP cell harvest

The spiked cells were recovered from the blood sample by running the “PXP_SMP_v1” protocol on the PP (Appendix S5.1). Like the PR1 cell harvest, the captured cells (~ 200 μL) were collected into a low binding Eppendorf tube. Subsequently, 0.2 μL of 1 mg·mL^−1^ of Hoechst dye was added to this tube. The content of this tube was then transferred to a poly‐l‐lysine‐coated well in a 96‐well plate, which was placed in an incubator (37 °C, 5% CO_2_) for 1 h to allow the cells to settle before visualization.

##### PP in‐cassette staining

The PP does not enable in‐cassette staining. However, there is an option to remove the cassette from the PP before proceeding to the cell harvest. At this step (see Appendix S5.2), the cassette was removed from the PP, and in‐cassette staining was performed on the PR1 as described in PR1 in‐cassette staining section (Fig. 1B).

### 2.3 Calculation of recovery rate from CTC isolation technologies

#### 2.3.1 Enumeration of recovered cells

**Fig. 2:**
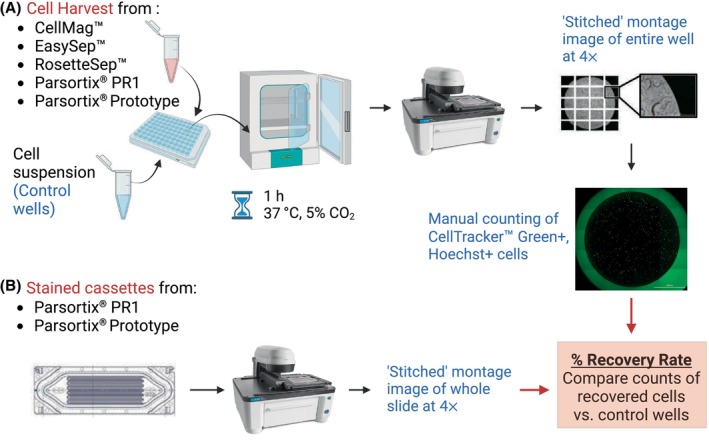
Calculation of recovery rate from circulating tumor cell (CTC) isolation technologies. (A) After CTC isolation, the recovered cells from the CellMag™, EasySep™, RosetteSep™, Parsortix® PR1, and Parsortix® Prototype (PP) were incubated for 1 h along with the associated control wells. Then, these wells were scanned using an imaging protocol (BioTek Lionheart FX‐automated microscope) to enumerate the total number of recovered cancer cells stained with Hoechst (DAPI channel) and CellTracker™ Green dye (GFP channel). (B) The PR1 and PP‐stained cassettes were visualized using another automated imaging protocol. The percentage recovery rate was calculated by comparing the count of recovered cells against the average cell count from the control wells. This figure was created with BioRender.com.

After the 1‐h incubation, the recovered cells collected in the 96‐well plate from the CellMag™, EasySep™, RosetteSep™, PR1, and PP (harvest) systems and their corresponding control wells were imaged using an automated imaging protocol and the BioTek Lionheart FX‐automated microscope (Agilent Technologies Inc., Santa Clara, CA, USA). A protocol was developed (using gen5 3.12 software, Agilent Technologies Inc.) capable of capturing images (tiles) from all areas of the well at a magnification of 4× (Fig. 2A, Fig. S1). This protocol divided the well into 6 × 4 image “tiles” which were then stitched together to create a montage of the entire well. These images were captured using a Z‐stack (10 focal planes) to ensure that spiked cells at different focal planes could be visualized and enumerated. This protocol was set up to capture images with the DAPI (Hoechst), GFP (CellTracker™ Green), and Bright Field channels. After capturing the montage image, the number of recovered cancer cells in each individual tile was manually counted. Cells that were positive for CellTracker™ Green and Hoechst, along with a morphologically intact nucleus, were counted as recovered cancer cells. For visualizing the CellMag™ harvest, the RFP and Cy5 channels were also included during the scanning of the entire well to assess the expression of cytokeratin and CD45, as per the kit's staining protocol.

From the PR1 and PP in‐cassette staining experiments, the cells captured and stained on the Parsortix® slides were visualized using a similar automated imaging protocol on the BioTek Lionheart FX‐automated microscope and gen5 3.12 software (Fig. 2B, Fig. S2). A second automated imaging protocol to scan the whole slide at 4× magnification with the DAPI (Hoechst), GFP (CKs, EpCAM), RFP (Vimentin), and CY5 (CD45) channels. This protocol divided the slide into 12 × 27 (rows × columns) image “tiles” which were then stitched together to create a montage of the entire slide. After capturing the montage image of the whole slide, the number of recovered cancer cells in each individual tile was manually counted. Cells that were positive for Hoechst, EpCAM/PanCK/CK19, and Vimentin, and negative for CD45, along with a morphologically intact nucleus, were counted as recovered cancer cells.

#### 2.3.2 Recovery rate

The percentage recovery rate of each CTC isolation technology was calculated according to the equation below:

$$\begin{array}{l} \text{The percentage recovery rate}\left(\%\mathrm{RR}\right)\\ \;=\begin{array}{l} \frac{\text{Number of recovered cancer cells counted}}{\text{Average number of cancer cells counted in control wells}}\\ \times 100. \end{array} \end{array}$$

### 2.4 Isolation of CTCs from the blood of lung cancer patients using the Parsortix® PR1

Between January 2023 and October 2023, patients with resectable NSCLC were prospectively recruited to the CLuB liquid biopsy study at St. James's Hospital Dublin. Ethical approval for the study was obtained from the St. James' Hospital (SJH)/Tallaght University Hospital (TUH) Joint Research Ethics Committee (JREC) (Ref. No. 041018/8804), in compliance with GDPR and Health Research Regulations 2018. The study was conducted in accordance with the precepts of the Code of Ethics of The World Medical Association (Declaration of Helsinki). All patients provided their appropriate written informed consent to participate in the study prior to enrollment. Following informed consent, peripheral blood samples were collected from lung cancer patients in 10 mL BD Vacutainer™ K2EDTA Tubes (VS367525; Becton, Dickinson and Company, Plymouth, UK). The isolation and enumeration of CTCs from patient blood samples were performed using the Parsortix® PR1 in‐cassette staining method as outlined in Section 2.2.4 and PR1 in‐cassette staining. CTCs were identified based on their staining profiles: epithelial CTCs (Hoechst+, PanCK/CK19/EpCAM+, Vim−, CD45−) or CTCs undergoing epithelial–mesenchymal transition (EMT CTCs; Hoechst+, PanCK/CK19/EpCAM+, Vim+, CD45−). Representative images were captured using the Leica SP8 confocal microscope (Leica Microsystems GmbH, Wetzlar, Germany) and processed using imagej software (U.S. National Institutes of Health, Bethesda, MD, USA).

### 2.5 Immunofluorescence staining

Cells were seeded into 96‐well plates at a density of 10 000 cells/well and allowed to adhere overnight at 37 °C with 5% CO_2_. After removing the culture media and washing the cells with 100 μL DPBS, the cells were fixed with 20 μL of 4% formaldehyde diluted in DPBS for 10 min at room temperature. The cells were then washed twice with 100 μL DPBS for 2 min to remove excess formaldehyde. Blocking was performed for 1 h at room temperature, followed by overnight staining with the fluorescent‐conjugated antibody and Hoechst nuclear dye at 4 °C in the dark. For intracellular staining of cytokeratins and vimentin, 0.3% Triton X‐100 was used as a permeabilization reagent in both the blocking buffer (5% BSA in DPBS) and the antibody dilution buffer (0.5% BSA in DPBS). Triton X‐100 was excluded from these buffers when staining for the transmembrane protein EpCAM. All reagents were the same as those mentioned in PR1 in‐cassette staining section. Following staining, the antibody/Hoechst solution was removed, and the cells were washed three times with 100 μL DPBS for 5 min each. Images of the cells were captured using the Lionheart FX‐automated microscope. If necessary, the background signal was subtracted from the images using imagej software.

### 2.6 Statistical analysis

Three independent spike‐in experiments were conducted on different days for each method to calculate the percentage recovery rate. The difference in recovery rates was analyzed by ANOVA with Tukey's *post hoc* test when comparing more than two variables. The unpaired *t* test (parametric, two‐tailed) was used to compare differences in recovery rates only between the CellMag™ and the Parsortix® PR1 (in‐cassette staining) technologies. Statistical analyses were performed using graphpad prism version 8.0.2 (Graphpad Software, Dotmatics, Boston, MA, USA). A *P* value of < 0.05 was considered significant.

## 3 Results

### 3.1 Recovery of H1975 cells across different CTC isolation technologies

**Fig. 3:**
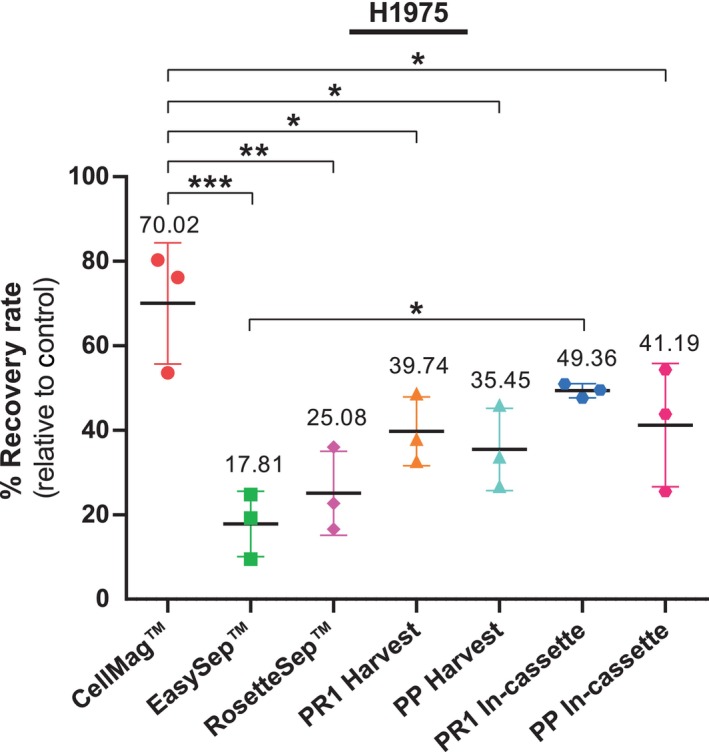
Comparison of the various circulating tumor cell (CTC) enrichment methods using the H1975 lung cancer cell line. A standardized spike‐in protocol was used to evaluate seven different CTC enrichment methods across five technologies: the CellMag™, EasySep™, RosetteSep™, Parsortix® PR1, and the Parsortix® Prototype (PP) systems. The differences in recovery rates between cell harvest versus in‐cassette staining for the Parsortix® (PR1 and PP) systems were also evaluated. The lines indicate the range of the percentage recovery rates (%RR) with standard deviation (error bars) observed in three independent spike‐in experiments for H1975 cells (*n* = 3). The mean %RR for each technology is also displayed. One‐way ANOVA followed by Tukey's multiple comparison test were used to compare recoveries between the seven different isolation methods using graphpad prism (version 8.0.2). Statistically significant differences in %RR are represented as follows: *P < 0.05, **P < 0.01, ***P < 0.001.

**Fig. 4:**
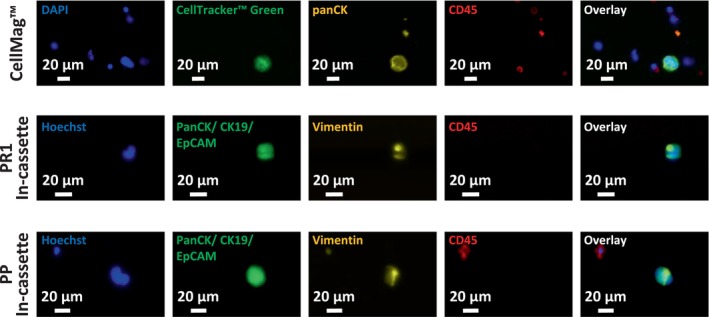
Representative staining images of spiked H1975 lung cancer cells enriched using different methods. Top panel: Approximately 100 H1975 cells (prelabeled with CellTracker™ Green) were spiked into 5 mL of whole blood and processed through CellMag™ system (*n* = 3). Recovered cells were stained with antibodies against cytokeratins (conjugated to phycoerythrin, yellow), CD45 (conjugated to allophycocyanin, red), and DAPI nuclear stain (blue). Middle and bottom panels: Approximately 100 H1975 cells were spiked into 5 mL of whole blood and recovered using the PR1 and PP in‐cassette staining, respectively (*n* = 3 for each method). Captured cells were stained with Alexa Fluor (AF)‐conjugated antibodies against Cytokeratins and EpCAM (AF488, green), Vimentin (AF546, yellow), CD45 (AF647, red), and Hoechst nuclear stain (blue). Representative images were captured using the BioTek Lionheart FX‐automated microscope and analyzed using imagej software. Scale bars represent 20 μm.

**Fig. 5:**
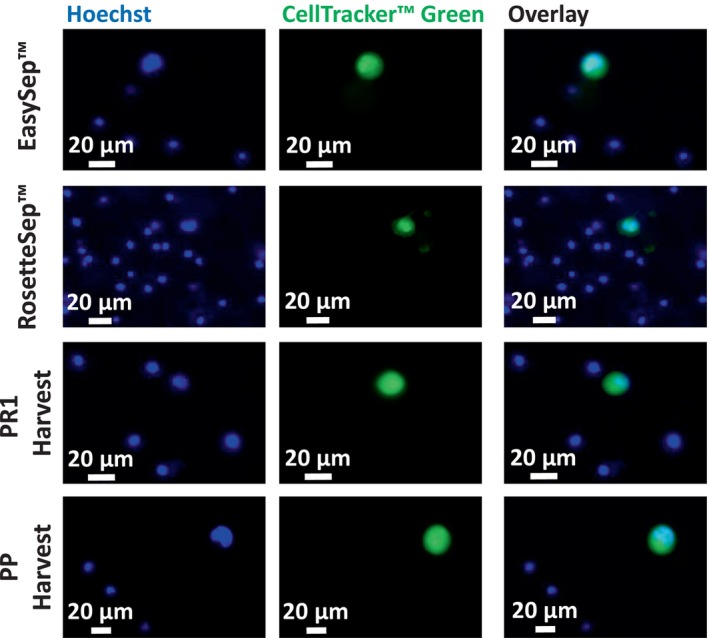
Representative staining images of spiked and prelabeled H1975 lung cancer cells enriched using different CTC isolation technologies. Approximately 100 H1975 cells (prelabeled with CellTracker™ Green) were spiked into 5 mL of whole blood and processed through the EasySep™, RosetteSep™, PR1 (harvest), and PP (harvest) methods (*n* = 3). The enriched cell suspension from each method was stained with Hoechst nuclear dye and the number of recovered cancer cells was counted to calculate recovery rate. Representative images were captured using the BioTek Lionheart FX‐automated microscope and analyzed using imagej software. Scale bars represent 20 μm.

H1975, a NSCLC line was initially used in spike‐in experiments to compare seven different CTC enrichment methods across five technologies. The mean percentage recovery rates (%RR) for each technology are presented in Fig. 3. The control well counts and the range of %RR, along with standard deviations observed across three independent spike‐in experiments for each technology, are presented in Table S1. Representative staining images of spiked H1975 lung cancer cells enriched using the seven different CTC isolation methods are displayed in Figs 4 and 5.

#### 3.1.1 CellMag™

As observed in Fig. 3, the CellMag™ system had the highest recovery rate of H1975 cells among all the methods tested with a mean of 70.02 ± 14.37%. This method had a significantly higher rate of recovery compared to all the other technologies, except the PR1 in‐cassette staining (Table S2). This was expected, as the CellMag™ system is based on the CellSearch®, which is the gold standard for EpCAM‐positive CTC isolation and FDA‐approved for certain cancers. The protocol was easy to follow, and the kit includes its own staining protocol, eliminating the need for antibody optimization. However, the process is very time‐consuming, involving many incubation steps and thus requiring significant “hands‐on” time (Table 1).

#### 3.1.2 EasySep™ and RosetteSep™

The EasySep™ and RosetteSep™ methods had the lowest recovery rates of 17.81 ± 7.74% and 25.08 ± 9.94%, respectively. Although no significant differences were found in the %RR values, whether normalized relative to the control well or not, narrower standard deviation values were observed with %RR relative to control (Table S1). Additionally, the difference between the %RR of EasySep™ and PR1 (in‐cassette staining) changed from not statistically significant (*P* = 0.2392, Table S3) to significant (*P* = 0.0271) when normalized (Table S2).

Both systems from StemCell Technologies capture CTCs by negative selection. As shown in Fig. 5, the RosetteSep™ method resulted in more leukocyte contamination compared to the EasySep™ method. Despite its low recovery rate, the EasySep™ system was the easiest and fastest to use. The advantage of both methods is their speed and suitability for downstream applications, such as CTC culture.

#### 3.1.3 Parsortix® PR1 and PP

The PR1 in‐cassette staining yielded the second highest recovery rate for H1975 cells (49.36 ± 1.67%). As shown in Fig. 3 and Table S1, the spike‐in results for PR1 in‐cassette staining were the most consistent among the three replicates compared to all the other technologies tested. Furthermore, the recovery of spiked cells using PR1 in‐cassette staining was significantly higher than with the EasySep™ method (*P* = 0.0271, Table S2). There was no significant difference in H1975 recovery rate between the PR1 and PP systems (in both cell harvest and in‐cassette staining) (*P* > 0.05). This was an anticipated result, as both systems utilize the same CTC isolation method. There was a trend of increased recovery rates for in‐cassette staining compared to the harvest protocol when using both the PR1 and PP systems, but these differences were not significant.

### 3.2 Determination of the optimal CTC enrichment technology for lung cancer

#### 3.2.1 Characterization of lung cancer cell lines by immunofluorescence staining

**Fig. 6:**
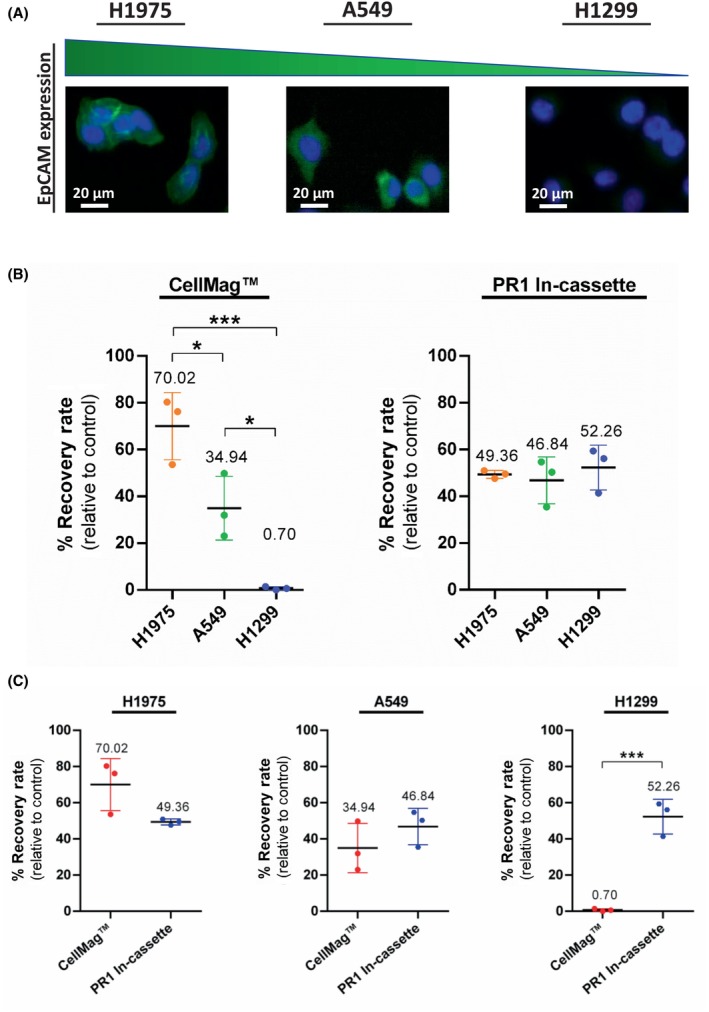
Comparison of the recovery rates of the CellMag™ system and Parsortix® PR1 in‐cassette staining method using H1975, A549, and H1299 lung cancer cell lines having different EpCAM expression levels. (A) Representative immunofluorescent staining images of EpCAM^high^ H1975, EpCAM^moderate^ A549, and EpCAM^low^ H1299 lung cancer cell lines stained with anti‐EpCAM antibody (AF488, green) and Hoechst nuclear dye (blue) (*n* = 3). Representative images were captured using the BioTek Lionheart FX‐automated microscope and analyzed using ImageJ software. Scale bars represent 20 μm. (B) Scatter dot plots comparing the recovery rates of H1975, A549, and H1299 cell lines using the CellMag™ and PR1 in‐cassette staining. The results are given as the mean ± SD. Asterisks indicate statistical significance between cell lines (*P < 0.05, ***P < 0.001, one‐way ANOVA followed by Tukey's test, *n* = 3). (C) Scatter dot plots comparing the recovery rates of CellMag™ and PR1 in‐cassette staining for each cell line. The results are given as the mean ± SD. Asterisks indicate statistical significance between CTC enrichment technologies (***P < 0.001, unpaired *t* test, *n* = 3). Statistical analysis was performed using graphpad prism (version 8.0.2).

To further investigate the optimal CTC enrichment technology for lung cancer, additional spike‐in experiments were conducted with A549 and H1299 cell lines using the CellMag™ and Parsortix® PR1 in‐cassette staining methods, which yielded the highest recovery rates with H1975 cells. These cell lines were selected based on their different EpCAM expression profiles. According to the Human Protein Atlas database, the normalized transcript per million (nTPM) values for EpCAM were as follows: H1975 (134.1), A549 (6.4), and H1299 (1.2) (Table S4) (https://www.proteinatlas.org). To confirm these data, immunofluorescent staining was performed to detect their EpCAM expression at protein level. As shown in Fig. 6A, EpCAM was highly expressed (EpCAM^high^) in H1975 cells and to a lesser extent (EpCAM^moderate^) in A549 cells. In contrast, H1299 cells had low EpCAM expression (EpCAM^low^). Moreover, the expression of other epithelial markers (various cytokeratins) and the mesenchymal marker Vimentin, targeted with corresponding antibodies used in the Parsortix® PR1 in‐cassette staining protocol, were also determined in these three cell lines by immunofluorescence staining (Fig. S3). While H1975 and A549 cell lines displayed both epithelial and mesenchymal characteristics, the H1299 cell line had remarkably high Vimentin expression compared to its low‐level expression of epithelial markers.

#### 3.2.2 Comparison of the recovery of lung cancer cell lines with different EpCAM expression levels using CellMag™ and Parsortix® PR1 in‐cassette staining technologies

Following validation of the EpCAM expression levels using immunofluorescence staining, the %RR of A549 and H1299 cells, spiked by following the same protocol as for H1975 cells, were determined using CellMag™ and Parsortix® PR1 in‐cassette staining technologies (Table S5, Fig. S4 and Table S6, Fig. S5, respectively).

In compliance with the detected EpCAM expression levels (Fig. 6A), the capturing capacity of the CellMag™ system were substantially reduced with EpCAM^moderate^ A549 cells and EpCAM^low^ H1299 cells compared to the EpCAM^high^ H1975 cell line (Fig. 6B). The %RRs of H1975, A549, and H1299 cells were 70.02 ± 14.37%, 34.94 ± 13.65%, and 0.70 ± 0.71%, respectively. There was a significant decrease in %RR between H1975 and A549 cells (*P* = 0.0221) and between A549 and H1299 cells (*P* = 0.0245) (Table S7). Furthermore, the CellMag™ system showed a very significant reduction in recovery rates between H1975 and H1299 cells (*P* = 0.0008).

In contrast, when the PR1 in‐cassette staining method was performed, the %RRs were relatively consistent among H1975 (49.36 ± 1.67%), A549 (46.84 ± 10.06%), and H1299 (52.26 ± 9.59%) cell lines, and there was no significant difference between their recoveries (Fig. 6B, Table S8). Remarkably, the recovery rate of H1299 cells using the PR1 in‐cassette staining method was significantly higher than the CellMag™ system (*P* = 0.0007; Fig. 6C, Table S9).

### 3.3 Investigation of the CTC isolation and detection ability of Parsortix® PR1 in‐cassette staining from patient blood

**Fig. 7:**
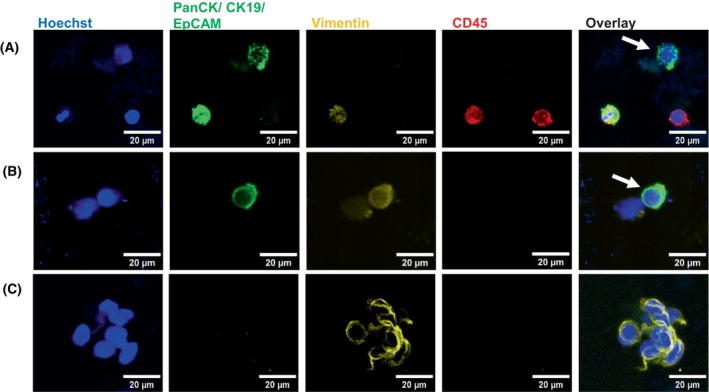
Representative confocal fluorescent microscopy images of single CTCs and a cell cluster isolated from the blood of lung cancer patients using Parsortix® PR1 in‐cassette staining. CTCs were isolated and detected from peripheral blood samples of lung cancer patients using the Parsortix® PR1 in‐cassette staining method (*n* = 2). Cells were stained with Alexa Fluor (AF)‐conjugated antibodies against Pan‐cytokeratin, Cytokeratin 19 and EpCAM (AF488, green), Vimentin (AF546, yellow) and CD45 (AF647, red), and Hoechst nuclear stain (blue). CTCs were identified based on their staining: (A) a single epithelial CTC (Hoechst+, PanCK/CK19/EpCAM+, Vim−, CD45−) as indicated by the white arrow; (B) a single CTC undergoing epithelial–mesenchymal transition (Hoechst+, PanCK/CK19/EpCAM+, Vim+, CD45−) as indicated by the white arrow; (C) a mesenchymal cell cluster (PanCK/CK19/EpCAM−, Vimentin+, CD45−). Representative images were captured using the Leica SP8 confocal microscope and processed using imagej software. Scale bars represent 20 μm.

The Parsortix® PR1 in‐cassette staining method was determined as optimal based on the results with three lung cancer cell lines displaying different epithelial and mesenchymal profiles. To validate these results obtained from lung cancer cell lines, the PR1 in‐cassette staining protocol was conducted with blood samples from lung cancer patients. As shown in Fig. 7, this method successfully captured single CTCs with either only epithelial characteristics (Fig. 7A) or both epithelial and mesenchymal characteristics (Fig. 7B). Furthermore, this method was also able to capture a mesenchymal cell cluster from a patient sample as shown in Fig. 7C.

## 4 Discussion

Isolating and identifying rare cells in the blood is akin to searching for a needle in a haystack. While various techniques have been developed for CTC isolation and enumeration, each technology comes with its own set of strengths and weaknesses (Table 1). Hence, comparative studies become particularly crucial, especially in scenarios where there is no FDA‐approved system for CTC detection in a specific cancer type, such as the case with lung cancer.

Initial spike‐in experiments with established cell lines are valuable for identifying the optimal CTC isolation and enumeration technology before handling precious patient samples. In these experiments, calculating the recovery rate based on the enumeration of cancer cells in healthy donor blood is a commonly employed analysis to assess the efficiency of these CTC technologies [19, 27, 28, 29].

The recovery rate of CTC enrichment technologies varies based on factors such as the size, morphology, and biomarker expression levels of the tested cell line, along with the compatibility of these features with the respective technique. The fact that the current gold standard, CellSearch®, is FDA‐approved only for metastatic breast, colorectal, and prostate cancers underscore this variability. Since there is currently no FDA‐approved CTC isolation and enumeration technology for lung cancer, we conducted a head‐to‐head comparison of seven different enrichment methods using five different technologies before working with samples from early‐stage lung cancer patients.

The H1975 lung adenocarcinoma cell line is known for its heterogeneous phenotype due to underdoing epithelial to mesenchymal transition (EMT) [27, 30], a characteristic observed in a high proportion of NSCLC patients and sometimes even among CTCs of individual lung cancer patients [30]. Therefore, the H1975 cell line, displaying different EpCAM expression levels within the same culture conditions, was selected to compare recovery rates of EpCAM‐dependent and ‐independent technologies. Since the CellSearch™ system is the gold standard for detecting EpCAM‐positive CTCs, we included its “research‐use only” version, CellMag™, as a positive control for comparison studies. Additionally, to our knowledge, this is the first study comparing the isolation efficacy of CellMag™ through spike‐in experiments involving cancer cells.

In this study, CellMag™ exhibited the highest recovery rate when tested with EpCAM‐positive H1975 cell line among the assessed technologies. Surprisingly, our literature review did not unveil any prior studies spiking lung cancer cells to assess the recovery rate of CellSearch®, despite its frequent utilization for CTC enumeration in lung cancer patients [31, 32, 33, 34, 35]. While we observed a commendable 70% recovery of heterogeneous, EpCAM^high^ H1975 lung cancer cell line with CellMag™, its advanced version, CellSearch®, was only able to detect ≥ 2 CTCs in 20% of the 168 lung cancer patients, as reported by Allard et al. [36]. Additionally, the capture rate with CellSearch® (32%) was lower compared to Parsortix® PR1 (harvest) (61%) when analyzing blood samples from 97 NSCLC patients in parallel [37]. The primary factor contributing to this diminished sensitivity in lung cancer patient samples is likely due to the missing of EpCAM‐negative CTCs with mesenchymal characteristics, which are associated with the tumor's metastatic potential [38], shorter progression‐free survival [39], and poorer survival [40] in lung cancer [41, 42].

To further investigate our hypothesis regarding the limitations of EpCAM‐dependent CTC enrichment systems, such as CellMag™, in lung cancer; we conducted spike‐in experiments with EpCAM^moderate^ A549 cells and EpCAM^low^ (considered as EpCAM‐negative) H1299 cells using the CellMag™ and Parsortix® PR1 in‐cassette staining systems. The data confirmed a significant decrease in the recovery of these cell lines compared to EpCAM^high^ H1975 cells when the EpCAM‐dependent CellMag™ system was used. Similarly, in a multicenter study conducted by the CANCER‐ID Consortium [27], the recovery of EpCAM^high^ NCI‐H441 and EpCAM^low^ NCI‐H1563 lung cancer cell lines was determined using five different CTC enrichment technologies: Siemens, VyCAP, RareCyte, Parsortix®, and CellSearch®. As expected, CellSearch® (as well as Parsortix® and Siemens) showed high recovery of EpCAM^high^ NCI‐H441 cells. Although all EpCAM‐independent technologies showed recovery rates ranging from 32% to 76% with EpCAM^low^ NCI‐H1563 cells, CellSearch® was unable to enrich them, similar to our results with EpCAM^low^ H1299 cells using the CellMag™.

Consequently, the exploration of marker‐independent CTC enrichment technologies becomes crucial, particularly for lung cancer cases [43]. Despite exhibiting lower recovery rates in spike‐in experiments with H1975 cells, size‐ and deformability‐based Parsortix® systems or negative depletion based RosetteSep™ and EasySep™ emerge as potential alternatives. Furthermore, the flexibility of using customizable antibodies for CTC enumeration allows for the selection of the markers of interest for each patient after enrichment with these technologies. This adaptability enhances the potential for a more personalized and effective approach in the context of lung cancer diagnostics and monitoring.

Among the seven different CTC enrichment methods tested, Parsortix® PR1 (in‐cassette staining) demonstrated the second highest recovery rate, coupled with the highest consistency between biological replicates with H1975 cells. Remarkably, the recovery of EpCAM^high^ H1975 cells was consistent with EpCAM^moderate^ A549 and EpCAM^low^ H1299 cell lines, demonstrating the capability of this system to detect CTCs independent of their phenotype. While the hands‐on time is minimal with semiautomated Parsortix® systems, the process of scanning the entire cassette for CTC enumeration could become time‐consuming when employing a manual microscope, as discussed later. The harvest option primarily addresses imaging issues encountered with in‐cassette staining and is advantageous for subsequent analyses like whole‐genome sequencing (WGS) [44], single‐cell RNA sequencing (scRNAseq) [45, 46], PCR [45, 47, 48], fluorescence *in situ* hybridization (FISH) [45], and culturing CTCs [49, 50]. Moreover, it is well suited for analyzing both circulating tumor DNA (ctDNA) and CTCs from the same blood tube [51]. However, harvest had slightly lower recovery rates compared to in‐cassette staining as observed with both Parsortix® systems (*P* > 0.05) which is in line with the trend shown in other studies [52, 53]. Parsortix® Prototype, the upgraded version of PR1 still under development, shortened the blood processing time and eliminated the need for flicking the blood tube to avoid unwanted clogging during enrichment. However, the recovery rate of PP was found slightly lower than PR1 with both harvest and in‐cassette staining protocols (*P* > 0.05) and requires PR1 for in‐cassette staining.

On the other hand, RosetteSep™ and EasySep™ serve as cost‐effective CTC enrichment technologies by eliminating labeled blood cells using density‐gradient centrifugation and magnetic fields, respectively. In this study, their recovery rates were found lower than those of CellMag™ and Parsortix® PR1 and PP systems when 100 cells were spiked. One possible explanation for their lower isolation efficiency could be the loss of some CTCs due to encapsulation within the unwanted blood cell clusters to be eliminated. However, instead of enumeration, these systems are particularly favorable for culturing CTCs. Especially RosetteSep™, both the human CD45 depletion cocktail and the CTC enrichment cocktail containing anti‐CD36, is widely used for culturing CTCs in lung cancer [54, 55, 56] as well as other cancer types [57, 58, 59]. However, EasySep™ is generally combined with another enrichment technology for culturing CTCs. Liao et al. [60] developed a two‐step enrichment protocol using EasySep™ human CD45 depletion kit followed by spheroid culture to isolate both epithelial and/or mesenchymal CTCs and cultured these with a success rate of 46% in blood samples of 13 head and neck cancer patients. In a later study, they also added the erythrocyte lysis step before EasySep™ and spheroid culture to isolate a better‐purified CTC population [61]. Interestingly, Liu et al. [62] discovered the highest recovery rate using only the EasySep™ human CD45 depletion kit (58%) after spiking 100 SW620 colon cancer cells into 5 mL of blood, compared to the recovery rates of using only the EasySep™ EpCAM(+) FITC‐positive selection kit (25%), or a combination of both (22.5%), which were similar to our 18% recovery rate with the H1975 cell line. Additionally, they successfully cultured and passaged CTCs from the pleural effusion of NSCLC patients as well as the ascetic fluids of colon and ovarian cancer patients using the depletion kit alone. Finally, RosetteSep™ [28, 63], CellSearch® [64], and Parsortix® PR1 [37, 65, 66] have shown the capability to capture CTC clusters, which are associated with poor prognosis in many cancer types, including lung cancer [67]. Similarly, a mesenchymal cell cluster in the blood of a lung cancer patient was detected by the Parsortix® PR1 in‐cassette staining method in this study.

As depicted earlier, there is considerable variability in recovery rates observed across different studies. Interestingly, this variability persists even when the same technology and cell lines are employed. Ntzifa et al. [68] spiked 10, 100, and 1000 H1975 cells into 10 mL of blood and assessed the recovery rate of Parsortix® PR1 based on *C*_t_ (cycle threshold or quantification cycle, *C*_q_) values for CK19 mRNA expression using qPCR, rather than enumeration via immunofluorescence imaging. Meanwhile, Obermayr et al. [48] enhanced the calculation of recovery rate by employing both qPCR and immunofluorescence imaging techniques. They introduced 100 H1975 cells into 18 mL of blood, resulting in a *C*_t_ value for CK19 of approximately ~ 19, in contrast to Ntzifa et al.'s *C*_t_ value of 34. Moreover, they achieved an average recovery rate of 80% based on immunofluorescence, surpassing our recovery rates with both in‐cassette (49%) and harvest (40%) protocols. These disparities in recovery rates among studies may stem from their enumeration protocol, which involves counting prestained fluorescent cells in‐cassette without fixation and antibody‐staining steps before harvesting them for qPCR analysis. In a comprehensive investigation, Papadaki et al. also spiked H1975 cells together with A549 and SK‐MES lung cancer cell lines (100 cells/5 mL) to compare semiautomated approaches ISET and Parsortix® with manual enrichment methods, namely density gradient medium and erythrocyte lysis buffer, along with their combined usage with CD45 depletion technology employing beads, Dynabeads [66]. Notably, direct air‐drying on lysine‐coated slides resulted in a higher recovery rate than cytospin (500 ***g*** for 2 min vs. 5 min) following cell harvesting. Interestingly, their recovery rate of H1975 cells postharvest with air‐drying on lysine‐coated slides (57 ± 11%) was found to be higher than our recovery rate with PR1 harvest (40 ± 8%). While the recovery of the SK‐MES cell line (57 ± 16%) was close to that of the H1975 cell line, the highest recovery rate (87 ± 5%) was observed with the A549 cell line, surpassing our A549 recovery using the PR1 in‐cassette staining method. In contrast, Cohen et al. [52] reported that the Parsortix® PR1 in‐cassette staining (capture) method recovered 51% of 100 spiked H1299 cells in 5 mL of blood with high linearity (*R* ^2^ = 0.9731), similar to our 52% H1299 recovery.

There are various parameters, including spiked cell number, blood volume, type of blood collection tube, time to process blood, imaging system, researcher's expertise especially in manual technologies, and the approach used for recovery rate calculation, that may contribute to these inconsistencies in the literature regarding the recovery rate of CTC isolation and enumeration techniques.

As there is no universally established protocol for spike‐in experiments, methodologies can vary among research groups and may not always be explicitly detailed in the literature. To achieve a cell suspension with a specific cell count, researchers commonly employ the direct serial dilution method [49] or combine this with a second counting step to determine the absolute spiked cell count in the blood tube [19] or readjust cell count with further dilution by including control wells [19, 66] before introducing healthy donor blood. In our study, we chose the serial dilution method and included control wells with an equivalent volume of the spiked‐in cell suspension for comparative analysis. The observed cell count in control wells varied around 100, sometimes falling below or exceeding that number. Consequently, we calculated the recovery rate as a percentage, based on the spiked cell count after isolation relative to the average cell count from these control wells. We conclude that incorporating control wells and subsequently normalizing the recovery rate relative to these wells in the serial dilution method can enhance accuracy, particularly when spiked cell numbers are low, such as 100 cells or below.

Spiked cell number is another critical parameter that can influence the recovery rate. Drucker et al. [28] conducted experiments where they spiked varying cell numbers (10, 100, 1000, and/or 10 000 cells) of the MDA‐MB‐231 breast cancer cell line to assess the isolation efficacy of DynaBeads®, EasySep™, RosetteSep™ (containing anti‐CD36 system), and ScreenCell®. While there were no significant changes in %RR for RosetteSep™ (34–40%) when 1000, 100, and 10 cells were spiked, interassay variability was significant when only 10 cells were spiked. Additionally, a significant difference in %RR was observed when reducing the spiked cell number from 10 000 to 1000 with both EasySep™ and Dynabeads CD45‐depletion technologies followed by an (EpCAM+)‐based second enrichment.

Testing a wider range of spiked cell numbers provides better insights into the linearity and detection limit for each technique's recovery rate. Moreover, the proximity of the spiked cell count to the CTC counts in the targeted patient population is crucial, especially when dealing with early‐stage cancer patients who may have potentially low CTC counts, ranging from 0 to 5 [23]. Although one limitation of this study is the fixed number of spiked cells at 100, this number enabled us to capture a broad spectrum of recovery rates across seven different enrichment methods and to highlight differences between these technologies more clearly. On the other hand, some of these technologies have already demonstrated consistent recovery rates with high linearity across a wide range of spiked cell numbers. For example, Parsortix® PR1 in‐cassette staining provided high linearity (*R* ^2^ = 0.9182–0.9731) with lung and breast cancer cell lines [52]. Additionally, harvesting with the FDA‐cleared Parsortix® PC1 system showed great linearity when blood samples were spiked with 2–100 cells of live and/or fixed SKBR3, MCF7, and Hs578T breast cancer cell lines (*R* ^**2**^ ≥ 0.94) [19].

Extra caution is necessary when utilizing fluorescent cell lines in spike‐in experiments. It is imperative to verify the homogeneity of fluorescent intensity within the cell population by employing a fluorescent microscope or a flow cytometer before initiating any spike‐in experiment, especially when utilizing “stable” cell lines expressing fluorescent proteins. This precaution is essential because some cells within this population, initially expressing fluorescent proteins, may lose the gene coding for the fluorescent protein while retaining the selection gene, such as an antibiotic‐resistance gene, over time (i.e., in later cell passages) [69]. On the contrary, prestaining a nonfluorescent cell line with a fluorescent dye for live cells is a common approach in spike‐in experiments [48, 66]. However, it is crucial to note that the fluorescent signal can be substantially low when cells are stained in the presence of serum. Additionally, optimizing staining conditions, such as determining the optimal dye concentration and labeling time, is a prerequisite before conducting spike‐in experiments. In our initial spike‐in experiments, we used the fluorescent dye CellTracker™ on the GFP‐expressing variant of the H1975 cell line to ensure uniformity of the fluorescent signal. In subsequent experiments where GFP‐expressing cell lines were unavailable, A549 and H1299 cells were successfully stained using our optimized CellTracker™ staining protocol.

Another crucial factor influencing the CTC enumeration process is the imaging system. Whether the system is manual or automated significantly impacts the reliability of recovery rate results and should not be underestimated. First, manual imaging requires heightened attention from researchers to avoid the inadvertent recounting of the same cell. On the other hand, automated imaging systems prove to be a substantial time‐saver compared to their manual counterparts. For instance, in Parsortix® PR1 and PP systems, employing in‐cassette staining can prolong the comprehensive scanning of the entire cassette using a manual fluorescent microscope. Moreover, addressing challenges arises when dealing with multiple planes within microfluidic cassettes for size‐based cell selection. In other size‐based CTC detection technologies employing filtration may encounter similar challenges due to the filters' having rough microscopic surfaces or difficulty distinguishing fluorescent signals when cancer and noncancer cells are captured on the same pore [70]. Consequently, these systems may require advanced imaging systems explicitly designed to automatically scan large areas quickly, operate at higher magnifications (e.g., 100×, 400×), and include Z‐stack imaging options. These advanced imaging systems are generally integrated with a CTC enumeration software, which is essential to facilitate cell imaging easily and standardize the enumeration process. However, it is worth noting that open‐source image analysis software, such as ACCEPT [71], specifically developed for identifying and counting CTCs, can also significantly enhance the analytical capabilities of researchers. In addition to adopting advanced imaging systems and software solutions, the recently introduced Portrait+ CTC staining kit and its customizable version, Portrait Flex, offer the flexibility to image a smaller area (comparable in size to one well of a 96‐well plate) on the unique cytospin slides included in the kits postharvesting by Parsortix® systems [72, 73].

Aside from CTC isolation technology, the variability in recovery rate is influenced by the researchers' familiarity with the technology, particularly in cases where manual handling is predominantly utilized. In this study, the standard deviation of recovery rates across three independent replicates for each CTC enrichment technology varied from 1.67% to 14.58%, consistent with published findings [27, 48, 49, 53, 66]. These variations fall within expected ranges and could be influenced by various factors such as the level of automation, differences among healthy blood donors, and even variations in cell passage numbers. For example, we noted enhanced recovery rates in the last replicates when employing manual CellMag™, EasySep™, and RosetteSep™ (Table S1). While there was no statistically significant difference in recovery rates between the semiautomated Parsortix® PR1 and PP systems with both harvest and in‐cassette protocols (*P* > 0.05), greater consistency in recovery rate values was observed with the commercialized Parsortix® PR1 using the in‐cassette protocol. Similarly, automated CellSearch® and prototype Siemens filtration unit provided higher reproducibility compared to Parsortix® PR1, VyCaP, and RareCyte [27]. As FDA‐approved CellSearch® provided the most reproducible results with proper documentation service, this highlights the need for developing an automated system, having a combined enrichment and staining protocol, an automatic microscopic scanning unit, and CTC analysis software, to implement CTC technologies in clinics.

## 5 Conclusions

In this study, a series of manual and automated CTC isolation technologies were compared for their efficiency in enriching CTCs. Initial spike‐in experiments using the H1975 cell line were conducted to evaluate the recovery rates of the following CTC enrichment technologies: CellMag™, EasySep™, RosetteSep™, Parsortix® PR1, and PP systems. Each method had its own advantages and disadvantages. The CellMag™ system and Parsortix® PR1 in‐cassette staining method yielded the highest recovery of EpCAM^high^ H1975 cells. However, the recovery rate of the EpCAM‐dependent CellMag™ system was significantly reduced with EpCAM^moderate^ A549 and EpCAM^low^ H1299 cell lines. The size‐ and deformability‐based Parsortix® PR1 in‐cassette staining method showed cell phenotype‐independent and consistent recovery rates among all lung cancer cell lines. Furthermore, this method was demonstrated to be capable of isolating different phenotypes of CTCs as either single cells or cell clusters from the blood samples of lung cancer patients. While further optimization and validation are required, the Parsortix® PR1 in‐cassette staining is considered optimal and may have a potential clinical application in lung cancer.

## Conflict of interest

The authors declare no conflict of interest.

## Author contributions

VMS, EO, and KG contributed to conceptualization and writing – original draft preparation. VMS and EO contributed to experiments. VMS, EO, MPW, and KG contributed to methodology. SH and GJF contributed to patient/donor consent and blood sample collection from healthy donors. VMS, EO, MPW, SPF, BDH, FL, JJOL, SOT, LOD, and KG contributed to writing – review and editing. LOD, KG, JJOL, and SOT contributed to funding acquisition. All authors have made substantial contributions to the acquisition and interpretation of data. All authors have approved the final version. Each author agreed to be accountable for all aspects of the work in ensuring that questions related to the accuracy or integrity of any part of the work are appropriately investigated and resolved.

## Supporting information


**Appendix S1.** Protocol for CellMag™ Epithelial CTC Kit.
**Appendix S2.** Protocol for EasySep™ Direct Human CTC Enrichment Kit.
**Appendix S3.** Protocol for RosetteSep™ Human CD45 Depletion Cocktail.
**Appendix S4.** Protocol for Parsortix® PR1.
**Appendix S4.1.** Protocol for Parsortix® PR1 in‐cassette staining.
**Appendix S4.2.** Protocol for Parsortix® PR1 cell harvest.
**Appendix S5.** Protocol for Parsortix® prototype (PP).
**Appendix S5.1.** Protocol for Parsortix® prototype (PP) cell harvest.
**Appendix S5.2.** Protocol for Parsortix® prototype (PP) in‐cassette staining.

**Fig. S1.** Automated imaging protocol for scanning a well of 96‐well plate at 4× to image cell harvest from CellMag™, EasySep™, RosetteSep™, Parsortix® PR1, and Parsortix® Prototype (PP) systems and the control wells.
**Fig. S2.** Automated imaging protocol for scanning the whole Parsortix® slides at 4× to image after Parsortix® PR1 and Parsortix® Prototype (PP) in‐cassette staining process.
**Fig. S3.** Representative immunofluorescent staining images of H1975, A549, and H1299 lung cancer cell lines.
**Fig. S4.** Representative staining images of spiked A549 lung cancer cells enriched using different methods.
**Fig. S5.** Representative staining images of spiked H1299 lung cancer cells enriched using different methods.

**Table S1.** Overview of the percentage of H1975 recovery rates from the seven different CTC enrichment methods tested in this study.
**Table S2.** Results of one‐way ANOVA followed by Tukey's multiple comparison test displaying the difference and significance in H1975 recovery rates (normalized to control).
**Table S3.** Results of one‐way ANOVA followed by Tukey's multiple comparison test displaying the difference and significance in H1975 recovery rates (not normalized to control).
**Table S4.** Normalized transcript per million (nTPM) values for each gene of interest in H1975, A549, and H1299 lung cancer cell lines extracted from Human Protein Atlas.
**Table S5.** Overview of the percentage of A549 recovery rates from the CellMag™ and Parsortix® PR1 in‐cassette staining methods.
**Table S6.** Overview of the percentage of H1299 recovery rates from the CellMag™ and Parsortix® PR1 in‐cassette staining methods.
**Table S7.** Results of one‐way ANOVA followed by Tukey's multiple comparison test displaying differences and significance in recovery rates of H1975, A549, and H1299 cell lines using the CellMag™ system.
**Table S8.** Results of one‐way ANOVA followed by Tukey's multiple comparison test displaying differences and significance in recovery rates of H1975, A549, and H1299 cell lines using the Parsortix® PR1 in‐cassette staining method.
**Table S9.** Results of unpaired *t* tests (parametric, two‐tailed) displaying differences and significance in recovery rates of H1975, A549, and H1299 cell lines between the CellMag™ system and Parsortix® PR1 in‐cassette staining method.

## Data Availability

The data that support the findings of this study are openly available in Figshare at http://doi.org/10.6084/m9.figshare.25146299 and http://doi.org/10.6084/m9.figshare.25146248.

## References

[1] Chemi F , Mohan S , Guevara T , Clipson A , Rothwell DG , Dive C . Early dissemination of circulating tumor cells: biological and clinical insights. Front Oncol. 2021;11:672195. 10.3389/fonc.2021.672195 34026650 PMC8138033

[2] de Miguel‐Pérez D , Bayarri‐Lara CI , Ortega FG , Russo A , Moyano Rodriguez MJ , Alvarez‐Cubero MJ , et al. Post‐surgery circulating tumor cells and AXL overexpression as new poor prognostic biomarkers in resected lung adenocarcinoma. Cancer. 2019;11:1750. 10.3390/cancers11111750 PMC6896005 31703465

[3] Hofman V , Ilie MI , Long E , Selva E , Bonnetaud C , Molina T , et al. Detection of circulating tumor cells as a prognostic factor in patients undergoing radical surgery for non‐small‐cell lung carcinoma: comparison of the efficacy of the CellSearch Assay™ and the isolation by size of epithelial tumor cell method. Int J Cancer. 2011;129:1651–1660. 10.1002/ijc.25819 21128227

[4] Li Z , Xu K , Tartarone A , Santarpia M , Zhu Y , Jiang G . Circulating tumor cells can predict the prognosis of patients with non‐small cell lung cancer after resection: a retrospective study. Transl Lung Cancer Res. 2021;10:995–1006. 10.21037/tlcr-21-149 33718038 PMC7947419

[5] Crosbie PA , Shah R , Krysiak P , Zhou C , Morris K , Tugwood J , et al. Circulating tumor cells detected in the tumor‐draining pulmonary vein are associated with disease recurrence after surgical resection of NSCLC. J Thorac Oncol. 2016;11:1793–1797. 10.1016/j.jtho.2016.06.017 27468936 PMC5063509

[6] Dong J , Zhu D , Tang X , Lu D , Qiu X , Li B , et al. Circulating tumor cells in pulmonary vein and peripheral arterial provide a metric for PD‐L1 diagnosis and prognosis of patients with non‐small cell lung cancer. PLoS One. 2019;14:e0220306. 10.1371/journal.pone.0220306 31348821 PMC6660086

[7] Hashimoto M , Tanaka F , Yoneda K , Takuwa T , Matsumoto S , Okumura Y , et al. Significant increase in circulating tumour cells in pulmonary venous blood during surgical manipulation in patients with primary lung cancer. Interact Cardiovasc Thorac Surg. 2014;18:775–783. 10.1093/icvts/ivu048 24618055

[8] Okumura Y , Tanaka F , Yoneda K , Hashimoto M , Takuwa T , Kondo N , et al. Circulating tumor cells in pulmonary venous blood of primary lung cancer patients. Ann Thorac Surg. 2009;87:1669–1675. 10.1016/j.athoracsur.2009.03.073 19463575

[9] Reddy RM , Murlidhar V , Zhao L , Grabauskiene S , Zhang Z , Ramnath N , et al. Pulmonary venous blood sampling significantly increases the yield of circulating tumor cells in early‐stage lung cancer. J Thorac Cardiovasc Surg. 2016;151:852–858. 10.1016/j.jtcvs.2015.09.126 26614417

[10] Hou JM , Krebs MG , Lancashire L , Sloane R , Backen A , Swain RK , et al. Clinical significance and molecular characteristics of circulating tumor cells and circulating tumor microemboli in patients with small‐cell lung cancer. J Clin Oncol. 2012;30:525–532. 10.1200/jco.2010.33.3716 22253462

[11] Krebs MG , Sloane R , Priest L , Lancashire L , Hou J‐M , Greystoke A , et al. Evaluation and prognostic significance of circulating tumor cells in patients with non–small‐cell lung cancer. J Clin Oncol. 2011;29:1556–1563. 21422424 10.1200/JCO.2010.28.7045

[12] Yang B , Qin A , Zhang K , Ren H , Liu S , Liu X , et al. Circulating tumor cells predict prognosis following tyrosine kinase inhibitor treatment in EGFR‐mutant non‐small cell lung cancer patients. Oncol Res. 2017;25:1601. 28474575 10.3727/096504017X14928634401178 PMC7840965

[13] Lindsay CR , Blackhall FH , Carmel A , Fernandez‐Gutierrez F , Gazzaniga P , Groen H , et al. EPAC‐lung: pooled analysis of circulating tumour cells in advanced non‐small cell lung cancer. Eur J Cancer. 2019;117:60–68. 31254940 10.1016/j.ejca.2019.04.019

[14] de Miguel‐Perez D , Ortega FG , Tejada RG , Martínez‐Única A , Peterson CB , Russo A , et al. Baseline extracellular vesicle miRNA‐30c and autophagic CTCs predict chemoradiotherapy resistance and outcomes in patients with lung cancer. Biomark Res. 2023;11:98. 37968730 10.1186/s40364-023-00544-y PMC10652484

[15] Eslami‐S Z , Cortés‐Hernández LE , Sinoquet L , Gauthier L , Vautrot V , Cayrefourcq L , et al. Circulating tumour cells and PD‐L1‐positive small extracellular vesicles: the liquid biopsy combination for prognostic information in patients with metastatic non‐small cell lung cancer. Br J Cancer. 2024;130:63–72. 37973956 10.1038/s41416-023-02491-9 PMC10781977

[16] Cohen SJ , Punt CJ , Iannotti N , Saidman BH , Sabbath KD , Gabrail NY , et al. Relationship of circulating tumor cells to tumor response, progression‐free survival, and overall survival in patients with metastatic colorectal cancer. J Clin Oncol. 2008;26:3213–3221. 10.1200/jco.2007.15.8923 18591556

[17] Cristofanilli M , Budd GT , Ellis MJ , Stopeck A , Matera J , Miller MC , et al. Circulating tumor cells, disease progression, and survival in metastatic breast cancer. N Engl J Med. 2004;351:781–791. 10.1056/NEJMoa040766 15317891

[18] de Bono JS , Scher HI , Montgomery RB , Parker C , Miller MC , Tissing H , et al. Circulating tumor cells predict survival benefit from treatment in metastatic castration‐resistant prostate cancer. Clin Cancer Res. 2008;14:6302–6309. 10.1158/1078-0432.Ccr-08-0872 18829513

[19] Templeman A , Miller MC , Cooke MJ , O'shannessy DJ , Gurung Y , Pereira T , et al. Analytical performance of the FDA‐cleared Parsortix® PC1 system. J Circ Biomark. 2023;12:26. 37601320 10.33393/jcb.2023.2629 PMC10434983

[20] Bankó P , Lee SY , Nagygyörgy V , Zrínyi M , Chae CH , Cho DH , et al. Technologies for circulating tumor cell separation from whole blood. J Hematol Oncol. 2019;12:48. 10.1186/s13045-019-0735-4 31088479 PMC6518774

[21] Bertoli E , De Carlo E , Basile D , Zara D , Stanzione B , Schiappacassi M , et al. Liquid biopsy in NSCLC: an investigation with multiple clinical implications. Int J Mol Sci. 2023;24:10803. 10.3390/ijms241310803 37445976 PMC10341684

[22] Hofman V , Bonnetaud C , Ilie MI , Vielh P , Vignaud JM , Fléjou JF , et al. Preoperative circulating tumor cell detection using the isolation by size of epithelial tumor cell method for patients with lung cancer is a new prognostic biomarker. Clin Cancer Res. 2011;17:827–835. 10.1158/1078-0432.Ccr-10-0445 21098695

[23] Wei T , Zhu D , Yang Y , Yuan G , Xie H , Shen R . The application of nano‐enrichment in CTC detection and the clinical significance of CTCs in non‐small cell lung cancer (NSCLC) treatment. PLoS One. 2019;14:e0219129. 10.1371/journal.pone.0219129 31344053 PMC6657845

[24] Fankhauser R , Chang M , Garrison Z , Berryman R , Lucero OM , Fuiten A , et al. Single‐cell identification of melanoma biomarkers in circulating tumor cells. Cancers. 2022;14:4921. 36230844 10.3390/cancers14194921 PMC9564060

[25] Cross SE , Jin YS , Rao J , Gimzewski JK . Nanomechanical analysis of cells from cancer patients. Nat Nanotechnol. 2007;2:780–783. 10.1038/nnano.2007.388 18654431

[26] Shaw Bagnall J , Byun S , Begum S , Miyamoto DT , Hecht VC , Maheswaran S , et al. Deformability of tumor cells versus blood cells. Sci Rep. 2015;5:18542. 10.1038/srep18542 26679988 PMC4683468

[27] Neves RPL , Ammerlaan W , Andree KC , Bender S , Cayrefourcq L , Driemel C , et al. Proficiency testing to assess technical performance for CTC‐processing and detection methods in CANCER‐ID. Clin Chem. 2021;67:631–641. 10.1093/clinchem/hvaa322 33491069

[28] Drucker A , Teh EM , Kostyleva R , Rayson D , Douglas S , Pinto DM . Comparative performance of different methods for circulating tumor cell enrichment in metastatic breast cancer patients. PLoS One. 2020;15:e0237308. 32790691 10.1371/journal.pone.0237308 PMC7425969

[29] Woo HJ , Kim S‐H , Kang HJ , Lee S‐H , Lee SJ , Kim JM , et al. Continuous centrifugal microfluidics (CCM) isolates heterogeneous circulating tumor cells via full automation. Theranostics. 2022;12:3676. 35664056 10.7150/thno.72511 PMC9131262

[30] Fachin F , Spuhler P , Martel‐Foley JM , Edd JF , Barber TA , Walsh J , et al. Monolithic chip for high‐throughput blood cell depletion to sort rare circulating tumor cells. Sci Rep. 2017;7:10936. 28883519 10.1038/s41598-017-11119-x PMC5589885

[31] Lindsay CR , Faugeroux V , Michiels S , Pailler E , Facchinetti F , Ou D , et al. A prospective examination of circulating tumor cell profiles in non‐small‐cell lung cancer molecular subgroups. Ann Oncol. 2017;28:1523–1531. 10.1093/annonc/mdx156 28633480

[32] Mondelo‐Macía P , García‐González J , León‐Mateos L , Anido U , Aguin S , Abdulkader I , et al. Clinical potential of circulating free DNA and circulating tumour cells in patients with metastatic non‐small‐cell lung cancer treated with pembrolizumab. Mol Oncol. 2021;15:2923–2940. 34465006 10.1002/1878-0261.13094 PMC8564635

[33] Tamminga M , Andree KC , Hiltermann TJN , Jayat M , Schuuring E , van den Bos H , et al. Detection of circulating tumor cells in the diagnostic leukapheresis product of non‐small‐cell lung cancer patients comparing CellSearch® and ISET. Cancers. 2020;12:896. 32272669 10.3390/cancers12040896 PMC7226321

[34] Truini A , Alama A , Dal Bello MG , Coco S , Vanni I , Rijavec E , et al. Clinical applications of circulating tumor cells in lung cancer patients by CellSearch system. Front Oncol. 2014;4:242. 10.3389/fonc.2014.00242 25237652 PMC4154446

[35] Wang X , Ma K , Yang Z , Cui J , He H , Hoffman AR , et al. Systematic correlation analyses of circulating tumor cells with clinical variables and tumor markers in lung cancer patients. J Cancer. 2017;8:3099–3104. 10.7150/jca.18032 28928901 PMC5604461

[36] Allard WJ , Matera J , Miller MC , Repollet M , Connelly MC , Rao C , et al. Tumor cells circulate in the peripheral blood of all major carcinomas but not in healthy subjects or patients with nonmalignant diseases. Clin Cancer Res. 2004;10:6897–6904. 10.1158/1078-0432.Ccr-04-0378 15501967

[37] Janning M , Kobus F , Babayan A , Wikman H , Velthaus J‐L , Bergmann S , et al. Determination of PD‐L1 expression in circulating tumor cells of NSCLC patients and correlation with response to PD‐1/PD‐L1 inhibitors. Cancers. 2019;11:835. 31212989 10.3390/cancers11060835 PMC6627043

[38] Zhang X , Wei L , Li J , Zheng J , Zhang S , Zhou J . Epithelial‐mesenchymal transition phenotype of circulating tumor cells is associated with distant metastasis in patients with NSCLC. Mol Med Rep. 2019;19:601–608. 10.3892/mmr.2018.9684 30483792

[39] Wang Y , Liu Y , Zhang L , Tong L , Gao Y , Hu F , et al. Vimentin expression in circulating tumor cells (CTCs) associated with liver metastases predicts poor progression‐free survival in patients with advanced lung cancer. J Cancer Res Clin Oncol. 2019;145:2911–2920. 10.1007/s00432-019-03040-9 31646374 PMC6861204

[40] Manjunath Y , Upparahalli SV , Avella DM , Deroche CB , Kimchi ET , Staveley‐O'Carroll KF , et al. PD‐L1 expression with epithelial mesenchymal transition of circulating tumor cells is associated with poor survival in curatively resected non‐small cell lung cancer. Cancers. 2019;11:806. 31212653 10.3390/cancers11060806 PMC6628040

[41] Okabe T , Togo S , Fujimoto Y , Watanabe J , Sumiyoshi I , Orimo A , et al. Mesenchymal characteristics and predictive biomarkers on circulating tumor cells for therapeutic strategy. Cancers. 2020;12:3588. 33266262 10.3390/cancers12123588 PMC7761066

[42] Pantazaka E , Vardas V , Roumeliotou A , Kakavogiannis S , Kallergi G . Clinical relevance of mesenchymal‐and stem‐associated phenotypes in circulating tumor cells isolated from lung cancer patients. Cancers. 2021;13:2158. 33947159 10.3390/cancers13092158 PMC8124761

[43] Hanssen A , Wagner J , Gorges TM , Taenzer A , Uzunoglu FG , Driemel C , et al. Characterization of different CTC subpopulations in non‐small cell lung cancer. Sci Rep. 2016;6:28010. 10.1038/srep28010 27302574 PMC4908396

[44] Acheampong E , Morici M , Abed A , Bowyer S , Asante D‐B , Lin W , et al. Powering single‐cell genomics to unravel circulating tumour cell subpopulations in non‐small cell lung cancer patients. J Cancer Res Clin Oncol. 2023;149:1941–1950. 10.1007/s00432-022-04202-y 35896898 PMC10097753

[45] Cohen EN , Jayachandran G , Moore RG , Cristofanilli M , Lang JE , Khoury JD , et al. A multi‐center clinical study to harvest and characterize circulating tumor cells from patients with metastatic breast cancer using the Parsortix® PC1 system. Cancers. 2022;14:5238. 36358657 10.3390/cancers14215238 PMC9656921

[46] Pillai SG , Siddappa CM , Ma C , Snider J , Kaushal M , Watson MA , et al. A microfluidic‐based filtration system to enrich for bone marrow disseminated tumor cells from breast cancer patients. PLoS One. 2021;16:e0246139. 10.1371/journal.pone.0246139 33989287 PMC8121342

[47] Markou AN , Londra D , Stergiopoulou D , Vamvakaris I , Potaris K , Pateras IS , et al. Preoperative mutational analysis of circulating tumor cells (CTCs) and plasma‐cfDNA provides complementary information for early prediction of relapse: a pilot study in early‐stage non‐small cell lung cancer. Cancers. 2023;15:1877. 36980762 10.3390/cancers15061877 PMC10047138

[48] Obermayr E , Koppensteiner N , Heinzl N , Schuster E , Holzer B , Fabikan H , et al. Cancer stem cell‐like circulating tumor cells are prognostic in non‐small cell lung cancer. J Pers Med. 2021;11:1225. 34834576 10.3390/jpm11111225 PMC8620949

[49] Hvichia G , Parveen Z , Wagner C , Janning M , Quidde J , Stein A , et al. A novel microfluidic platform for size and deformability based separation and the subsequent molecular characterization of viable circulating tumor cells. Int J Cancer. 2016;138:2894–2904. 26789903 10.1002/ijc.30007 PMC5069649

[50] Xu L , Mao X , Imrali A , Syed F , Mutsvangwa K , Berney D , et al. Optimization and evaluation of a novel size based circulating tumor cell isolation system. PLoS One. 2015;10:e0138032. 10.1371/journal.pone.0138032 26397728 PMC4580600

[51] Wishart G , Templeman A , Hendry F , Miller K , Pailhes‐Jimenez A‐S . Molecular profiling of circulating tumour cells and circulating tumour DNA: complementary insights from a single blood sample Utilising the Parsortix® system. Curr Issues Mol Biol. 2024;46:773–787. 38248352 10.3390/cimb46010050 PMC10814787

[52] Cohen EN , Jayachandran G , Hardy MR , Venkata Subramanian AM , Meng X , Reuben JM . Antigen‐agnostic microfluidics‐based circulating tumor cell enrichment and downstream molecular characterization. PLoS One. 2020;15:e0241123. 33095819 10.1371/journal.pone.0241123 PMC7584183

[53] Weidele K , Stojanović N , Feliciello G , Markiewicz A , Scheitler S , Alberter B , et al. Microfluidic enrichment, isolation and characterization of disseminated melanoma cells from lymph node samples. Int J Cancer. 2019;145:232–241. 30586191 10.1002/ijc.32092

[54] Hodgkinson CL , Morrow CJ , Li Y , Metcalf RL , Rothwell DG , Trapani F , et al. Tumorigenicity and genetic profiling of circulating tumor cells in small‐cell lung cancer. Nat Med. 2014;20:897–903. 10.1038/nm.3600 24880617

[55] Kapeleris J , Kulasinghe A , Warkiani ME , Oleary C , Vela I , Leo P , et al. Ex vivo culture of circulating tumour cells derived from non‐small cell lung cancer. Transl Lung Cancer Res. 2020;9:1795–1809. 33209602 10.21037/tlcr-20-521 PMC7653113

[56] Lee H‐L , Chiou J‐F , Wang P‐Y , Lu L‐S , Shen C‐N , Hsu H‐L , et al. Ex vivo expansion and drug sensitivity profiling of circulating tumor cells from patients with small cell lung cancer. Cancers. 2020;12:3394. 33207745 10.3390/cancers12113394 PMC7696848

[57] Brungs D , Minaei E , Piper A‐K , Perry J , Splitt A , Carolan M , et al. Establishment of novel long‐term cultures from EpCAM positive and negative circulating tumour cells from patients with metastatic gastroesophageal cancer. Sci Rep. 2020;10:539. 31953491 10.1038/s41598-019-57164-6 PMC6968999

[58] Gao D , Vela I , Sboner A , Iaquinta PJ , Karthaus WR , Gopalan A , et al. Organoid cultures derived from patients with advanced prostate cancer. Cell. 2014;159:176–187. 25201530 10.1016/j.cell.2014.08.016 PMC4237931

[59] Kulasinghe A , Perry C , Warkiani ME , Blick T , Davies A , O'Byrne K , et al. Short term ex‐vivo expansion of circulating head and neck tumour cells. Oncotarget. 2016;7:60101. 27517751 10.18632/oncotarget.11159 PMC5312371

[60] Liao C‐J , Hsieh C‐H , Wang H‐M , Chou W‐P , Chiu T‐K , Chang J‐H , et al. Isolation of label‐free and viable circulating tumour cells (CTCs) from blood samples of cancer patients through a two‐step process: negative selection‐type immunomagnetic beads and spheroid cell culture‐based cell isolation. RSC Adv. 2017;7:29339–29349.

[61] Liao C‐J , Hsieh C‐H , Hung F‐C , Wang H‐M , Chou W‐P , Wu M‐H . The integration of a three‐dimensional spheroid cell culture operation in a circulating tumor cell (CTC) isolation and purification process: a preliminary study of the clinical significance and prognostic role of the CTCs isolated from the blood samples of head and neck cancer patients. Cancers. 2019;11:783. 31174311 10.3390/cancers11060783 PMC6627984

[62] Liu Z , Fusi A , Klopocki E , Schmittel A , Tinhofer I , Nonnenmacher A , et al. Negative enrichment by immunomagnetic nanobeads for unbiased characterization of circulating tumor cells from peripheral blood of cancer patients. J Transl Med. 2011;9:1–8. 10.1186/1479-5876-9-70 PMC3119001 21595914

[63] Funaki S , Sawabata N , Nakagiri T , Shintani Y , Inoue M , Kadota Y , et al. Novel approach for detection of isolated tumor cells in pulmonary vein using negative selection method: morphological classification and clinical implications. Eur J Cardiothorac Surg. 2011;40:322–327. 21215651 10.1016/j.ejcts.2010.11.029

[64] Costa C , Muinelo‐Romay L , Cebey‐López V , Pereira‐Veiga T , Martínez‐Pena I , Abreu M , et al. Analysis of a real‐world cohort of metastatic breast cancer patients shows circulating tumor cell clusters (CTC‐clusters) as predictors of patient outcomes. Cancers. 2020;12:1111. 32365530 10.3390/cancers12051111 PMC7281711

[65] Acheampong E , Abed A , Morici M , Spencer I , Beasley AB , Bowyer S , et al. Evaluation of PD‐L1 expression on circulating tumour cells in small‐cell lung cancer. Transl Lung Cancer Res. 2022;11:440–451. 35399573 10.21037/tlcr-21-819 PMC8988079

[66] Papadaki MA , Sotiriou AI , Vasilopoulou C , Filika M , Aggouraki D , Tsoulfas PG , et al. Optimization of the enrichment of circulating tumor cells for downstream phenotypic analysis in patients with non‐small cell lung cancer treated with anti‐PD‐1 immunotherapy. Cancers. 2020;12:1556. 32545559 10.3390/cancers12061556 PMC7352396

[67] Murlidhar V , Reddy RM , Fouladdel S , Zhao L , Ishikawa MK , Grabauskiene S , et al. Poor prognosis indicated by venous circulating tumor cell clusters in early‐stage lung cancers. Cancer Res. 2017;77:5194–5206. 28716896 10.1158/0008-5472.CAN-16-2072 PMC5600850

[68] Ntzifa A , Strati A , Kallergi G , Kotsakis A , Georgoulias V , Lianidou E . Gene expression in circulating tumor cells reveals a dynamic role of EMT and PD‐L1 during osimertinib treatment in NSCLC patients. Sci Rep. 2021;11:2313. 33504904 10.1038/s41598-021-82068-9 PMC7840727

[69] Chambers CB , Halford WP , Geltz J , Villamizar O , Gross J , Embalabala A , et al. A system for creating stable cell lines that express a gene of interest from a bidirectional and regulatable herpes simplex virus type 1 promoter. PLoS One. 2015;10:e0122253. 10.1371/journal.pone.0122253 25823013 PMC4378986

[70] Pailler E , Oulhen M , Billiot F , Galland A , Auger N , Faugeroux V , et al. Method for semi‐automated microscopy of filtration‐enriched circulating tumor cells. BMC Cancer. 2016;16:1–15. 10.1186/s12885-016-2461-4 PMC4946105 27417942

[71] Zeune L , van Dalum G , Decraene C , Proudhon C , Fehm T , Neubauer H , et al. Quantifying HER‐2 expression on circulating tumor cells by ACCEPT. PLoS One. 2017;12:e0186562. 10.1371/journal.pone.0186562 29084234 PMC5662084

[72] Portrait Flex. ANGLE plc website. [cited 2024 Jul 29]. Available from: https://angleplc.com/laboratory‐services/portrait‐flex/

[73] Portrait+ CTC staining kit. ANGLE plc website. [cited 2024 Jul 29]. Available from: https://angleplc.com/portrait‐plus/

