# Supplementary material for: A comparative study of circulating tumor cell isolation and enumeration technologies in lung cancer
Source: Mol Oncol. 2024 Aug 6;19(7):2014–37. doi: 10.1002/1878-0261.13705 (PMC12234383; doi:10.1002/1878-0261.13705)
Supplement: Supplementary file 2 — Fig. S1. Automated imaging protocol for scanning a well of 96‐well plate at 4× to image cell harvest from CellMag™, EasySep™, RosetteSep™, Parsortix® PR1, and Parsortix® Prototype (PP) systems and the control wells. Fig. S2. Automated imaging protocol for scanning the whole Parsortix® slides at 4× to image after Parsortix® PR1 and Parsortix® Prototype (PP) in‐cassette staining process. Fig. S3. Representative immunofluorescent staining images of H1975, A549, and H1299 lung cancer cell lines. Fig. S4. Representative staining images of spiked A549 lung cancer cells enriched using different methods. Fig. S5. Representative staining images of spiked H1299 lung cancer cells enriched using different methods. [file MOL2-19-2014-s001.pdf]

SUPPORTING INFORMATION

A comparative study of circulating tumor cell isolation and enumeration technologies in lung cancer

Volga M Saini[1,2,3,a], Ezgi Oner[1,2,3,a], Mark P Ward[3,4,5], Sinead Hurley[1,2,3,4], Brian David Henderson[3,4,5], Faye Lewis[3,4,5], Stephen P Finn[1,3,5], Gerard J Fitzmaurice[6], John J O'Leary[3,5], Sharon O'Toole[3,4,5], Lorraine O'Driscoll[3,7,8], Kathy Gately[1,2,3,*]

1 Thoracic Oncology Research Group, Trinity Translational Medicine Institute, St James's Hospital, Dublin, Ireland.
2 Department of Clinical Medicine, School of Medicine, Trinity College Dublin, Ireland.
3 Trinity St. James's Cancer Institute, Trinity College Dublin, Ireland.
4 Department of Obstetrics and Gynaecology, School of Medicine, Trinity College Dublin, Ireland.
5 Department of Histopathology and Morbid Anatomy, School of Medicine, Trinity College Dublin, Ireland.
6 Department of Cardiothoracic Surgery, St James's Hospital, Dublin, Ireland.
7 School of Pharmacy and Pharmaceutical Sciences, Trinity College Dublin, Ireland.
8 Trinity Biomedical Sciences Institute, Trinity College Dublin, Ireland.

a V.M.S. and E.O. should be considered joint first author.

*Corresponding author: Kathy Gately. Email: gatelyk@tcd.ie. Address: Thoracic Oncology Research Group, Department of Clinical Medicine, Trinity Translational Medicine Institute, St James's Hospital, Dublin, D08 W9RT, Ireland.

Supplementary Figures

Fig. S1. Automated imaging protocol for scanning a well of 96-well plate at 4X to image cell harvest from CellMag™, EasySep™, RosetteSep™, Parsortix® PR1 and Parsortix® Prototype (PP) systems and the control wells.

Fig. S2. Automated imaging protocol for scanning the whole Parsortix® slides at 4X to image after Parsortix® PR1 and Parsortix® Prototype (PP) in-cassette staining process.

Fig. S3. Representative immunofluorescent staining images of H1975, A549 and H1299 lung cancer cell lines.

Fig. S4. Representative staining images of spiked A549 lung cancer cells enriched using different methods.

Fig. S5. Representative staining images of spiked H1299 lung cancer cells enriched using different methods.

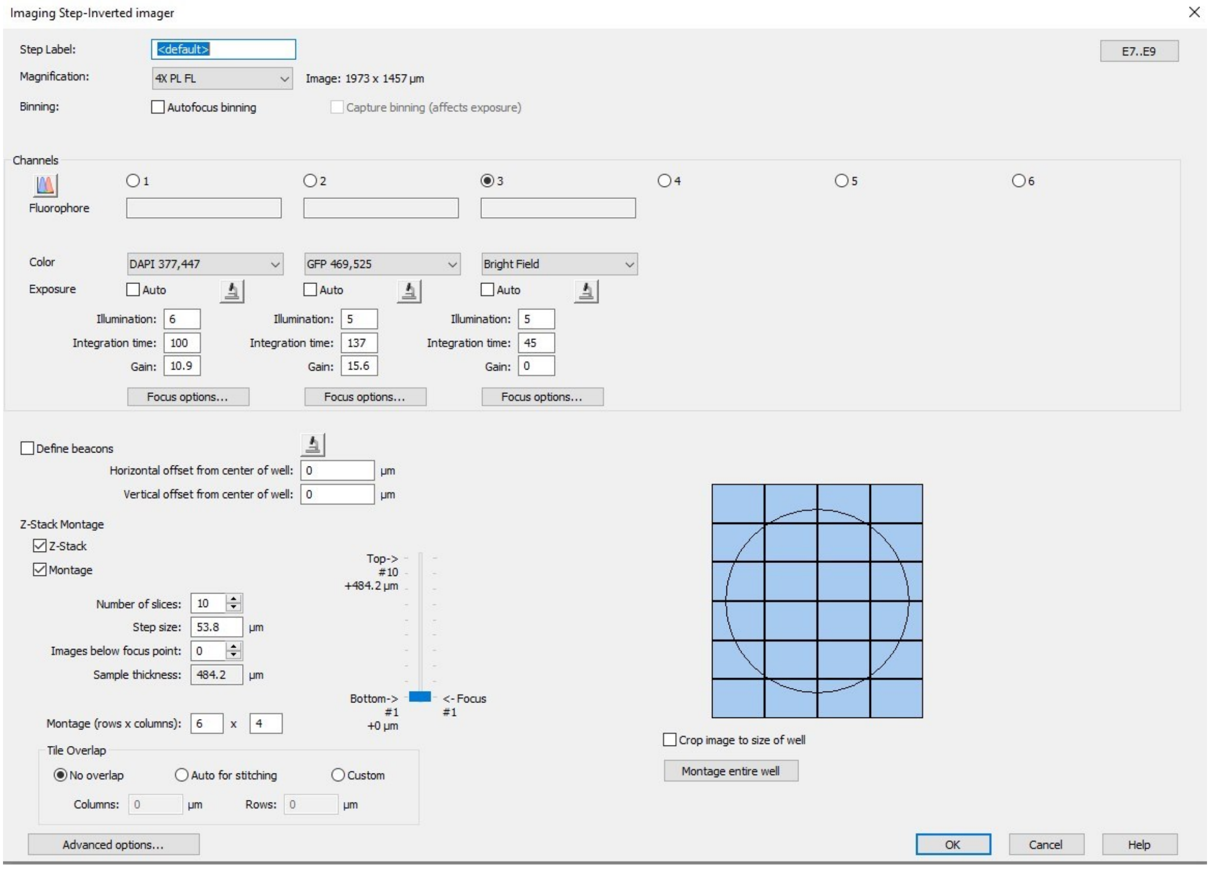

Fig. S1. Automated imaging protocol for scanning a well of 96-well plate at 4X to image cell harvest from CellMag™, EasySep™, RosetteSep™, Parsortix® PR1 and Parsortix® Prototype (PP) systems and the control wells. The recovered cells (in the 96 well plate) from the CellMag™, EasySep™, RosetteSep™, PR1 and PP (Harvest) systems and their respective control wells were imaged using an automated imaging protocol and the BioTek Lionheart FX automated microscope. The above protocol (Gen5 3.12 software) was established which can take images (tiles) from all areas of the well at a magnification of 4X. In this protocol, we divided the well into 6x4 image 'tiles' which were then 'stitched together' to create a 'montage' of the entire well. These images were taken using a Z-stack (10 focal planes). This protocol was set up to take images with the DAPI (Hoechst), GFP (CellTracker Green) and Bright Field channels. The Lionheart FX protocol file can be found on FigShare under the DOI: http://doi.org/10.6084/m9.figshare.25146299

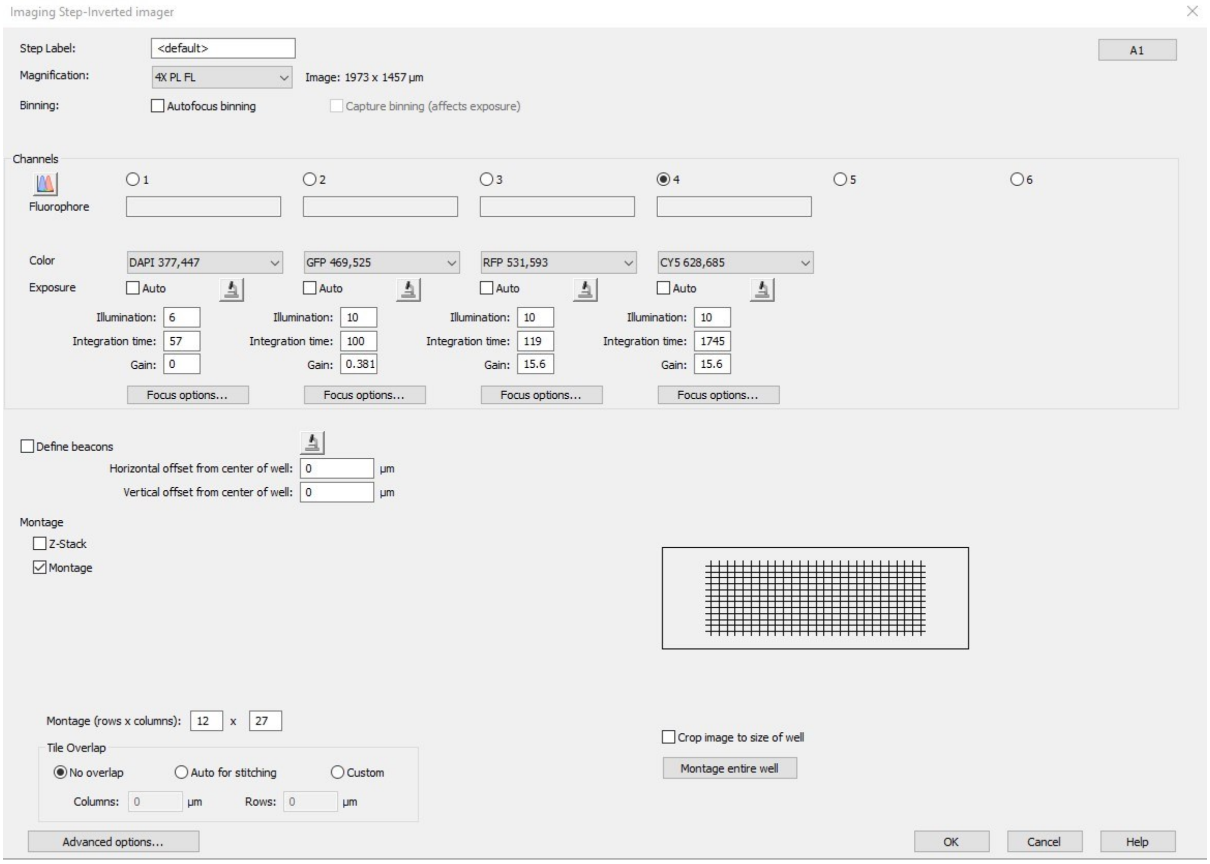

Fig. S2. Automated imaging protocol for scanning the whole Parsortix® slides at 4X to image after Parsortix® PR1 and Parsortix® Prototype (PP) in-cassette staining process. From the PR1 and PP in-cassette staining experiments, the spiked cells captured and stained on the Parsortix® slides were visualised using an automated imaging protocol on the BioTek Lionheart FX automated microscope and Gen5 3.12 software. The above protocol was used to scan the whole slide at 4X with the DAPI (Hoechst), GFP (CellTracker™ Green, CK, EpCAM), RFP (Vimentin), CY5 (CD45) channels. The slide was divided into 12x27 (rows x columns) image 'tiles' which were then 'stitched together' to create a 'montage' of the entire slide. The Lionheart FX protocol file can be found on FigShare under the DOI: http://doi.org/10.6084/m9.figshare.25146248

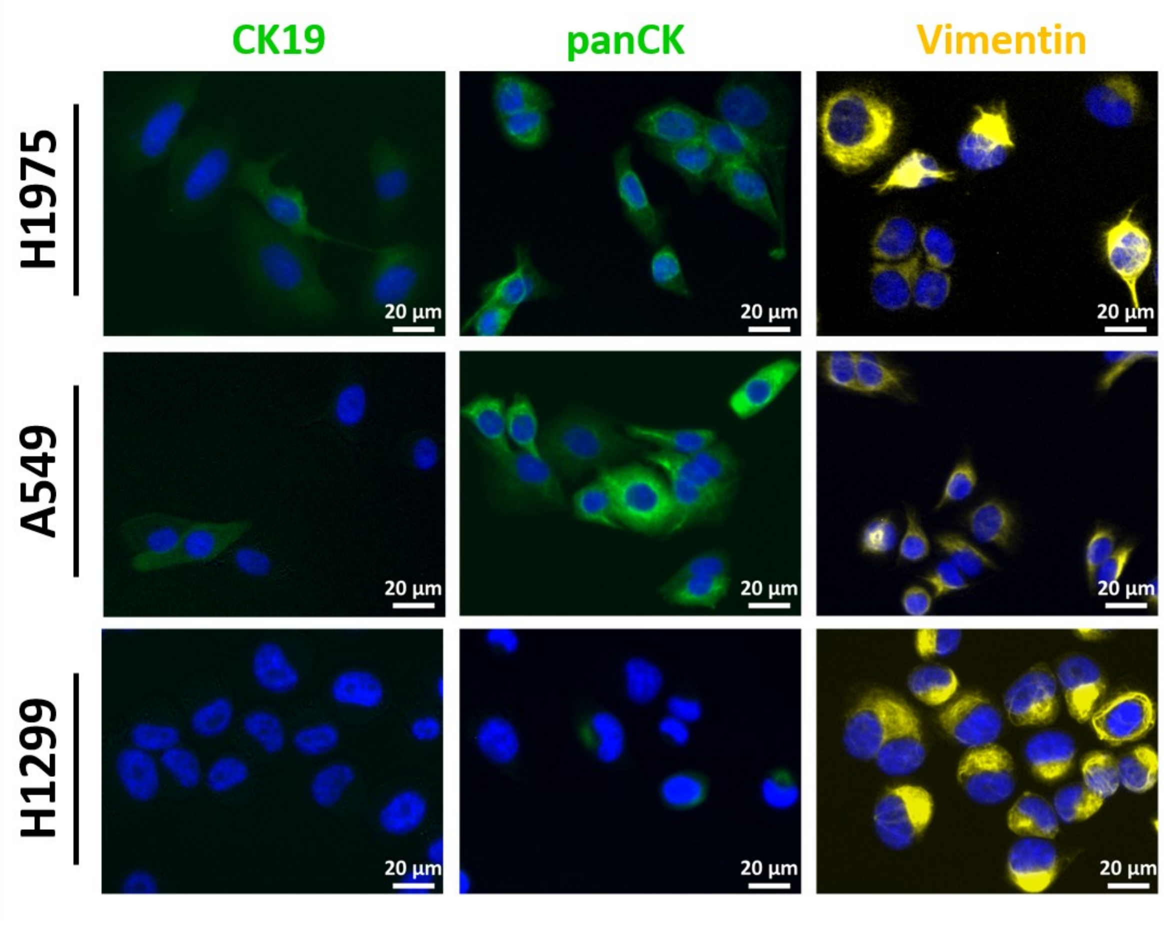

Fig. S3. Representative immunofluorescent staining images of H1975, A549 and H1299 lung cancer cell lines (n=3). Cells were stained with Alexa Fluor (AF)-conjugated antibodies against pan-cytokeratin (CK 4, 5, 6, 8, 10, 13 and 18), CK19 and EpCAM (AF488, green) and Vimentin (AF546, yellow), and Hoechst nuclear dye (blue). Representative images were captured using the BioTek Lionheart FX automated microscope and analysed using ImageJ software. Scale bars represent 20 µm.

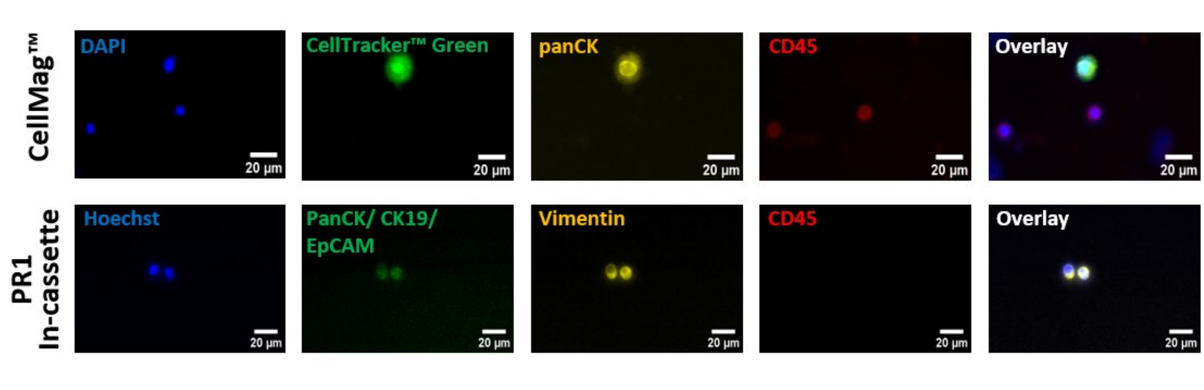

Fig. S4. Representative staining images of spiked A549 lung cancer cells enriched using different methods. *Top Panel:* Recovered pre-labeled A549 cells (with CellTracker™ Green) from the CellMag™ system. According to the kit's protocol, recovered cells were stained with antibodies against cytokeratins (conjugated to phycoerythrin, yellow), CD45 (conjugated to allophycocyanin, red) and DAPI nuclear stain (blue). *Bottom Panel:* A549 cells recovered using the PR1 in-cassette staining. Captured cells were stained with Alexa Fluor (AF)-conjugated antibodies against Cytokeratins and EpCAM (AF488, green), Vimentin (AF546, yellow), CD45 (AF647, red), and Hoechst nuclear stain (blue). Representative images were captured using the BioTek Lionheart FX automated microscope and analysed using ImageJ software. Scale bars represent 20 µm.

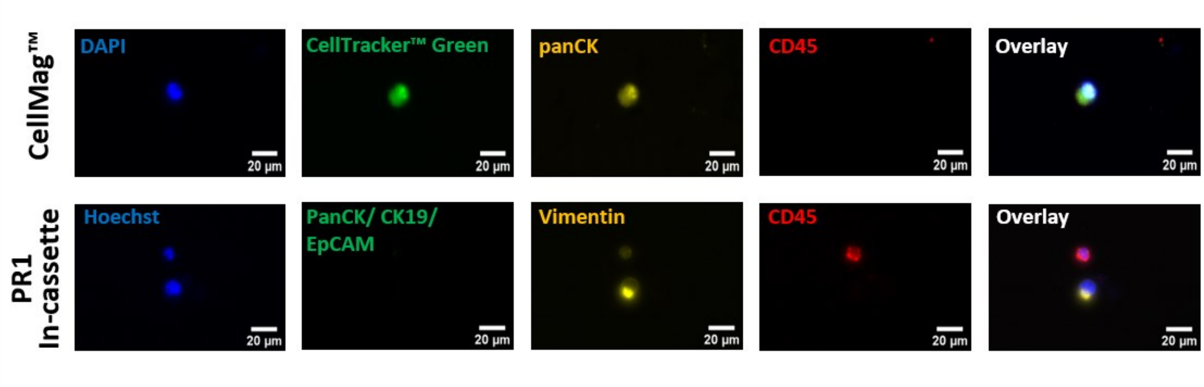

Fig. S5. Representative staining images of spiked H1299 lung cancer cells enriched using different methods. *Top Panel:* Recovered pre-labeled H1299 cells (with CellTracker™ Green) from the CellMag™ system. According to the kit's protocol, recovered cells were stained with antibodies against cytokeratins (conjugated to phycoerythrin, yellow), CD45 (conjugated to allophycocyanin, red) and DAPI nuclear stain (blue). *Bottom Panel:* H1299 cells recovered using the PR1 in-cassette staining. Captured cells were stained with Alexa Fluor (AF)-conjugated antibodies against Cytokeratins and EpCAM (AF488, green), Vimentin (AF546, yellow), CD45 (AF647, red), and Hoechst nuclear stain (blue). Representative images were captured using the BioTek Lionheart FX automated microscope and analysed using ImageJ software. Scale bars represent 20 µm.
