# Supplementary material for: A comparative study of circulating tumor cell isolation and enumeration technologies in lung cancer
Source: Mol Oncol. 2024 Aug 6;19(7):2014–37. doi: 10.1002/1878-0261.13705 (PMC12234383; doi:10.1002/1878-0261.13705)
Supplement: Supplementary file 3 — Table S1. Overview of the percentage of H1975 recovery rates from the seven different CTC enrichment methods tested in this study. Table S2. Results of one‐way ANOVA followed by Tukey's multiple comparison test displaying the difference and significance in H1975 recovery rates (normalized to control). Table S3. Results of one‐way ANOVA followed by Tukey's multiple comparison test displaying the difference and significance in H1975 recovery rates (not normalized to control). Table S4. Normalized transcript per million (nTPM) values for each gene of interest in H1975, A549, and H1299 lung cancer cell lines extracted from Human Protein Atlas. Table S5. Overview of the percentage of A549 recovery rates from the CellMag™ and Parsortix® PR1 in‐cassette staining methods. Table S6. Overview of the percentage of H1299 recovery rates from the CellMag™ and Parsortix® PR1 in‐cassette staining methods. Table S7. Results of one‐way ANOVA followed by Tukey's multiple comparison test displaying differences and significance in recovery rates of H1975, A549, and H1299 cell lines using the CellMag™ system. Table S8. Results of one‐way ANOVA followed by Tukey's multiple comparison test displaying differences and significance in recovery rates of H1975, A549, and H1299 cell lines using the Parsortix® PR1 in‐cassette staining method. Table S9. Results of unpaired t tests (parametric, two‐tailed) displaying differences and significance in recovery rates of H1975, A549, and H1299 cell lines between the CellMag™ system and Parsortix® PR1 in‐cassette staining method. [file MOL2-19-2014-s002.pdf]

SUPPORTING INFORMATION

A comparative study of circulating tumor cell isolation and enumeration technologies in lung cancer

Volga M Saini[1,2,3,a], Ezgi Oner[1,2,3,a], Mark P Ward[3,4,5], Sinead Hurley[1,2,3,4], Brian David Henderson[3,4,5], Faye Lewis[3,4,5], Stephen P Finn[1,3,5], Gerard J Fitzmaurice[6], John J O'Leary[3,5], Sharon O'Toole[3,4,5], Lorraine O'Driscoll[3,7,8], Kathy Gately[1,2,3,*]

1 Thoracic Oncology Research Group, Trinity Translational Medicine Institute, St James's Hospital, Dublin, Ireland.
2 Department of Clinical Medicine, School of Medicine, Trinity College Dublin, Ireland.
3 Trinity St. James's Cancer Institute, Trinity College Dublin, Ireland.
4 Department of Obstetrics and Gynaecology, School of Medicine, Trinity College Dublin, Ireland.
5 Department of Histopathology and Morbid Anatomy, School of Medicine, Trinity College Dublin, Ireland.
6 Department of Cardiothoracic Surgery, St James's Hospital, Dublin, Ireland.
7 School of Pharmacy and Pharmaceutical Sciences, Trinity College Dublin, Ireland.
8 Trinity Biomedical Sciences Institute, Trinity College Dublin, Ireland.

a V.M.S. and E.O. should be considered joint first author.

*Corresponding author: Kathy Gately. Email: gatelyk@tcd.ie. Address: Thoracic Oncology Research Group, Department of Clinical Medicine, Trinity Translational Medicine Institute, St James's Hospital, Dublin, D08 W9RT, Ireland.

Supplementary Tables

Table S1. Overview of the percentage of H1975 recovery rates from the 7 different CTC enrichment methods tested in this study.

Table S2. Results of one-way ANOVA followed by Tukey's multiple comparison test displaying the difference and significance in H1975 recovery rates (normalized to control).

Table S3. Results of one-way ANOVA followed by Tukey's multiple comparison test displaying the difference and significance in H1975 recovery rates (not normalized to control).

Table S4. Normalized transcript per million (nTPM) values for each gene of interest in H1975, A549 and H1299 lung cancer cell lines extracted from Human Protein Atlas.

Table S5. Overview of the percentage of A549 recovery rates from the CellMag™ and Parsortix® PR1 in-cassette staining methods.

Table S6. Overview of the percentage of H1299 recovery rates from the CellMag™ and Parsortix® PR1 in-cassette staining methods.

Supplementary Tables *(continue)*

Table S7. Results of one-way ANOVA followed by Tukey's multiple comparison test displaying differences and significance in recovery rates of H1975, A549 and H1299 cell lines using the CellMag™ system.

Table S8. Results of one-way ANOVA followed by Tukey's multiple comparison test displaying differences and significance in recovery rates of H1975, A549 and H1299 cell lines using the Parsortix® PR1 in-cassette staining method.

Table S9. Results of unpaired t-tests (parametric, two-tailed) displaying differences and significance in recovery rates of H1975, A549 and H1299 cell lines between the CellMag™ system and Parsortix® PR1 in-cassette staining method.

Table S1. Overview of the percentage of H1975 recovery rates from the 7 different CTC enrichment methods tested in this study.

CTC Enrichment Technology	Experimental repeats	Cell Counts						%Recovery Rate (relative to control)	%Recovery Rate (relative to control)	
		Control Well 1	Control Well 2	Control Well 3	Control wells		Recovered Cells			
					Mean	SD			Mean	SD
CellMag™	n=1	144	145	142	143.67	1.53	77	53.60%	70.02%	14.37%
	n=2	137	155	153	148.33	9.87	113	76.18%		
	n=3	125	111	134	123.33	11.59	99	80.27%		
EasySep™	n=1	78	91	N/A	84.5	9.19	8	9.47%	17.81%	7.74%
	n=2	123	119	133	125	7.21	24	19.20%		
	n=3	99	110	94	101	8.19	25	24.75%		
RosetteSep™	n=1	78	91	N/A	84.5	9.19	14	16.57%	25.08%	9.94%
	n=2	126.5	136	121	127.83	7.59	29	22.69%		
	n=3	123	119	133	125	7.21	45	36.00%		
PR1 Harvest	n=1	95	95	N/A	95	0.00	36	37.89%	39.74%	8.15%
	n=2	99	110	94	101	8.19	33	32.67%		
	n=3	170	135	139	148	19.16	72	48.65%		
PP Harvest	n=1	95	95	N/A	95	0.00	32	33.68%	35.45%	9.73%
	n=2	99	110	94	101	8.19	27	26.73%		
	n=3	170	135	139	148	19.16	68	45.95%		
PR1 In-cassette	n=1	104	106	N/A	105	1.41	50	47.62%	49.36%	1.67%
	n=2	100	110	N/A	105	7.07	52	49.52%		
	n=3	117	95	N/A	106	15.56	54	50.94%		
PP In-cassette	n=1	104	106	N/A	105	1.41	46	43.81%	41.19%	14.58%
	n=2	100	110	N/A	105	7.07	57	54.29%		
	n=3	117	95	N/A	106	15.56	27	25.47%		

Table S2. Results of one-way ANOVA followed by Tukey's multiple comparison test displaying the difference and significance in H1975 recovery rates (normalized to control)[a]

Tukey's multiple comparison test	Mean Diff.	95.00% CI of diff.	Significant?	Summary	Adjusted P Value[b]
CellMag™ vs. EasySep™	52.21	23.52 to 80.90	Yes	***	0.0004
CellMag™ vs. RosetteSep™	44.93	16.24 to 73.62	Yes	**	0.0015
CellMag™ vs. PR1 Harvest	30.28	1.585 to 58.97	Yes	*	0.0357
CellMag™ vs. PP Harvest	34.56	5.869 to 63.26	Yes	*	0.0141
CellMag™ vs. PR1 In-cassette	20.66	-8.038 to 49.35	No	ns	0.2454
CellMag™ vs. PP In-cassette	28.83	0.1320 to 57.52	Yes	*	0.0486
EasySep™ vs. RosetteSep™	-7.280	-35.97 to 21.41	No	ns	0.9721
EasySep™ vs. PR1 Harvest	-21.93	-50.62 to 6.765	No	ns	0.1948
EasySep™ vs. PP Harvest	-17.65	-46.34 to 11.05	No	ns	0.4027
EasySep™ vs. PR1 In-cassette	-31.55	-60.25 to -2.859	Yes	*	0.0271
EasySep™ vs. PP In-cassette	-23.38	-52.08 to 5.311	No	ns	0.1479
RosetteSep™ vs. PR1 Harvest	-14.65	-43.34 to 14.04	No	ns	0.6014
RosetteSep™ vs. PP Harvest	-10.37	-39.06 to 18.33	No	ns	0.8697
RosetteSep™ vs. PR1 In-cassette	-24.27	-52.97 to 4.421	No	ns	0.1242
RosetteSep™ vs. PP In-cassette	-16.10	-44.80 to 12.59	No	ns	0.5016
PR1 Harvest vs. PP Harvest	4.283	-24.41 to 32.98	No	ns	0.9983
PR1 Harvest vs. PR1 In-cassette	-9.623	-38.32 to 19.07	No	ns	0.9030
PR1 Harvest vs. PP In-cassette	-1.453	-30.15 to 27.24	No	ns	> 0.9999
PP Harvest vs. PR1 In-cassette	-13.91	-42.60 to 14.79	No	ns	0.6531
PP Harvest vs. PP In-cassette	-5.737	-34.43 to 22.96	No	ns	0.9916
PR1 In-cassette vs. PP In-cassette	8.170	-20.52 to 36.86	No	ns	0.9522

a Analysis was performed using GraphPad Prism (version 8.0.2).

b The p-value < 0.05 was considered statistically significant.

Table S3. Results of one-way ANOVA followed by Tukey's multiple comparison test displaying the difference and significance in H1975 recovery rates (not normalized to control)[a]

Tukey's multiple comparison test	Mean Diff.	95.00% CI of diff.	Significant?	Summary	Adjusted P Value[b]
CellMag™ vs. EasySep™	77.33	31.81 to 122.9	Yes	***	0.0007
CellMag™ vs. RosetteSep™	67.00	21.48 to 112.5	Yes	**	0.0027
CellMag™ vs. PR1 Harvest	49.33	3.810 to 94.86	Yes	*	0.0299
CellMag™ vs. PP Harvest	54.00	8.476 to 99.52	Yes	*	0.0158
CellMag™ vs. PR1 In-cassette	44.33	-1.190 to 89.86	No	ns	0.0586
CellMag™ vs. PP In-cassette	53.00	7.476 to 98.52	Yes	*	0.0182
EasySep™ vs. RosetteSep™	-10.33	-55.86 to 35.19	No	ns	0.9839
EasySep™ vs. PR1 Harvest	-28.00	-73.52 to 17.52	No	ns	0.4025
EasySep™ vs. PP Harvest	-23.33	-68.86 to 22.19	No	ns	0.5974
EasySep™ vs. PR1 In-cassette	-33.00	-78.52 to 12.52	No	ns	0.2392
EasySep™ vs. PP In-cassette	-24.33	-69.86 to 21.19	No	ns	0.5537
RosetteSep™ vs. PR1 Harvest	-17.67	-63.19 to 27.86	No	ns	0.8297
RosetteSep™ vs. PP Harvest	-13.00	-58.52 to 32.52	No	ns	0.9516
RosetteSep™ vs. PR1 In-cassette	-22.67	-68.19 to 22.86	No	ns	0.6267
RosetteSep™ vs. PP In-cassette	-14.00	-59.52 to 31.52	No	ns	0.9327
PR1 Harvest vs. PP Harvest	4.667	-40.86 to 50.19	No	ns	0.9998
PR1 Harvest vs. PR1 In-cassette	-5.000	-50.52 to 40.52	No	ns	0.9997
PR1 Harvest vs. PP In-cassette	3.667	-41.86 to 49.19	No	ns	> 0.9999
PP Harvest vs. PR1 In-cassette	-9.667	-55.19 to 35.86	No	ns	0.9885
PP Harvest vs. PP In-cassette	-1.000	-46.52 to 44.52	No	ns	> 0.9999
PR1 In-cassette vs. PP In-cassette	8.667	-36.86 to 54.19	No	ns	0.9935

a Analysis was performed using GraphPad Prism (version 8.0.2).

b The p-value < 0.05 was considered statistically significant.

Table S4. Normalized transcript per million (nTPM) values for each gene of interest in H1975, A549 and H1299 lung cancer cell lines extracted from Human Protein Atlas.

Lung Cancer Type	Cell line	nTPM values according to Human Protein Atlas database[a,b]												
		Epithelial markers										Mesenchymal markers		Leukocyte marker
		CK4	CK5	CK6A	CK7	CK8	CK10	CK13	CK18	CK19	EpCAM	Vimentin	N-cadherin (CDH2)	CD45 (PTPRC)
Adenocarcinoma	H1975	0.1	0.7	0.1	1440.6	1098.3	16.2	0.3	878.8	732.3	134.1	1695.1	0.2	1.3
Adenocarcinoma	A549	18.7	0.7	0.4	2936.5	4783.6	26.3	0.3	4951.8	194.8	6.4	1099.7	55.1	0.1
Large Cell Carcinoma	H1299	0	0.8	0.1	1.4	209.8	7.9	0.2	554.2	2.4	1.2	3012.9	12.5	0.4

a The nTPM values were extracted from Human Protein Atlas database (https://www.proteinatlas.org).

b The markers, which were targeted by antibodies used in this study, were written in bold.

Table S5. Overview of the percentage of A549 recovery rates from the CellMag™ and Parsortix® PR1 in-cassette staining methods.

CTC Enrichment Technology	Experimental repeats	Cell Counts						%Recovery Rate (relative to control)	%Recovery Rate (relative to control)	
		Control Well 1	Control Well 2	Control Well 3	Control wells		Recovered Cells			
					Mean	SD			Mean	SD
CellMag™	n=1	79	107	97	94.33	14.19	47	49.82%	34.94%	13.65%
	n=2	91	111	117	106.33	13.61	34	31.97%		
	n=3	122	114	103	113	9.54	26	23.01%		
PR1 In-cassette	n=1	126	127	120	124.33	3.79	68	54.69%	46.84%	10.06%
	n=2	118	118	119	118.33	0.58	42	35.49%		
	n=3	89	107	114	103.33	12.90	52	50.32%		

Table S6. Overview of the percentage of H1299 recovery rates from the CellMag™ and Parsortix® PR1 in-cassette staining methods.

CTC Enrichment Technology	Experimental repeats	Cell Counts							%Recovery Rate (relative to control)	%Recovery Rate (relative to control)	
		Control Well 1	Control Well 2	Control Well 3	Control Well 4	Control wells		Recovered Cells			
						Mean	SD			Mean	SD
CellMag™	n=1	129	159	120	152	140.00	18.49	2	1.43%	0.70%	0.71%
	n=2	115	102	100	N/A	105.67	8.14	0	0.00%		
	n=3	150	145	157	N/A	150.67	6.03	1	0.66%		
PR1 In-cassette	n=1	132	125	112	N/A	123.00	10.15	69	56.10%	52.26%	9.59%
	n=2	146	136	152	127	140.25	11.03	58	41.35%		
	n=3	147	124	135	140	136.50	9.68	81	59.34%		

Table S7. Results of one-way ANOVA followed by Tukey's multiple comparison test displaying differences and significance in recovery rates of H1975, A549 and H1299 cell lines using the CellMag™ system[a].

Tukey's multiple comparison test	Mean Diff.	95.00% CI of diff.	Significant?	Summary	Adjusted P Value[b]
A549 vs. H1975	-35.08	-63.76 to -6.406	Yes	*	0.0221
H1299 vs. H1975	-69.32	-98.00 to -40.64	Yes	***	0.0008
H1299 vs. A549	-34.24	-62.91 to -5.559	Yes	*	0.0245

a Analysis was performed using GraphPad Prism (version 8.0.2).

b The p-value < 0.05 was considered statistically significant.

Table S8. Results of one-way ANOVA followed by Tukey's multiple comparison test displaying differences and significance in recovery rates of H1975, A549 and H1299 cell lines using the Parsortix® PR1 in-cassette staining method[a].

Tukey's multiple comparison test	Mean Diff.	95.00% CI of diff.	Significant?	Summary	Adjusted P Value[b]
A549 vs. H1975	-2.527	-22.78 to 17.72	No	ns	0.9234
H1299 vs. H1975	2.903	-17.35 to 23.15	No	ns	0.9005
H1299 vs. A549	5.430	-14.82 to 25.68	No	ns	0.7038

a Analysis was performed using GraphPad Prism (version 8.0.2).

b The p-value < 0.05 was considered statistically significant.

Table S9. Results of unpaired t-tests (parametric, two-tailed) displaying differences and significance in recovery rates of H1975, A549 and H1299 cell lines between the CellMag™ system and Parsortix® PR1 in-cassette staining method.

CellMag™ vs PR1 in-cassette staining	Difference between means ± SEM	95% CI	P value	P value summary
H1975	-20.66 ± 8.348	-43.84 to 2.522	0.0686	ns
A549	11.90 ± 9.790	-15.28 to 39.08	0.2910	ns
H1299	51.57 ± 5.552	36.15 to 66.98	0.0007	***

a Analysis was performed using GraphPad Prism (version 8.0.2).

b The p-value < 0.05 was considered statistically significant.
